# Development of a novel adherence scale for antidepressants in pregnancy: Results from a cross-sectional study

**DOI:** 10.1016/j.rcsop.2026.100704

**Published:** 2026-01-12

**Authors:** Milica Zugic, Natasa Pejic, Saeed Hayati, Marleen M.H.J. van Gelder, Hedvig Nordeng

**Affiliations:** a Pharmacoepidemiology and Drug Safety Research Group, Department of Pharmacy, Faculty of Mathematics and Natural Sciences, University of Oslo, Sem Sælands vei 3, 0371 Oslo, Norway; b IQ Health Science Department, Radboud University Medical Center, Geert Grooteplein Zuid 10, 6525 GA Nijmegen, the Netherlands; c UiORealArt Convergence Environment, University of Oslo, Oslo, Norway; d Department of Child Health and Development, Norwegian Institute of Public Health; Lovisenberggata 8, 0456 Oslo, Norway

**Keywords:** Adherence, Antidepressants, Pregnancy, Psychometric scale, MAMP-AD, Validity

## Abstract

**Introduction:**

Adherence to antidepressant pharmacotherapy is essential for optimal treatment. Its assessment during pregnancy is challenging, as existing self-report scales are not tailored to pregnancy.

**Aim:**

This study aimed to develop a self-report scale to assess antidepressant adherence in pregnancy and evaluate its psychometric properties.

**Methods:**

This cross-sectional study used an anonymous online questionnaire administered in Norway between February and March 2022. Participants were pregnant or up-to-12-month postpartum individuals who had used antidepressants during pregnancy. *The Medication Adherence Measurement in Pregnancy for Antidepressants* (MAMP-AD) scale was developed based on prior research, expert input, and piloting. Exploratory factor analysis (EFA) was used to explore its underlying structure. Reliability was assessed using Cronbach's α. Construct validity was evaluated by examining associations between MAMP-AD scores and depressive and anxiety symptoms, and medication-related beliefs.

**Results:**

Ninety participants who used antidepressants during pregnancy completed the MAMP-AD scale. The final 15-item MAMP-AD scale had a score range of 0 to 48, with higher scores indicating higher adherence. EFA identified three distinct factors: 1) Minimizing exposure, 2) Barriers to antidepressant use, and 3) Benefits of antidepressant use. These explained 81.7% of the total variance in the MAMP-AD scale. The scale showed acceptable reliability (Cronbach's α = 0.80). Higher MAMP-AD scores were negatively associated with depression severity, supporting construct validity.

**Conclusion:**

The MAMP-AD is a newly developed, self-report scale for measuring antidepressant adherence in pregnancy, showing preliminary evidence of acceptable reliability and validity. Further research is needed to confirm the scale's psychometric properties and clinical utility.

## 1 Introduction

Depression and anxiety are among the most common psychiatric disorders during pregnancy, affecting approximately 18%^1^ and 15%^2^ of pregnant individuals, respectively. First-line treatment options include psychotherapy and antidepressant medication. Selective serotonin reuptake inhibitors are the most commonly prescribed antidepressants during pregnancy, with a global prevalence of around 3%.^3^ In Scandinavia, prevalence rates vary, reported at 1.8% in Norway, 3.7% in Denmark and Sweden, and as high as 7.0% in Iceland.^4^ Continuing antidepressant treatment during pregnancy has been associated with a reduced risk of relapse and may decrease the likelihood of postpartum depressive symptoms.^5^^,^^6^ However, concerns about fetal safety may cause patients to reduce or discontinue their antidepressant treatment during pregnancy.

Medication adherence, defined as “the process by which patients take their medication as prescribed”,^7^ is essential for the effective treatment of depression. Studies have shown that patients frequently discontinue their antidepressant treatment prematurely,^8^ with adherence rates of less than 50%.^9^^,^^10^ This issue warrants particular attention in pregnant populations, given that only 10% of those treated with antidepressants before pregnancy continue the medication into the third trimester, compared to 35% in the non-pregnant population over a similar period.^11^ **Table S1** provides an overview of prior studies on antidepressant adherence during pregnancy, showing both low adherence rates and the use of diverse methods to measure adherence.

Methods used to measure medication adherence include clinician assessments, patient self-reports, pill counts, plasma level measurements, and electronic medication event monitoring systems.^12^ While existing self-report adherence scales can offer valuable insights into attitudes, behaviors, and intentions, they often fail to address the unique challenges faced during pregnancy. Key factors such as fear of fetal exposure, perceived treatment benefits, and the presence of depressive symptoms can substantially influence adherence during pregnancy.^13^^,^^14^ Yet, these factors are insufficiently captured by currently available methods.^15^ As a result, adherence during pregnancy remains inadequately studied.

A reliable, pregnancy-specific measure of adherence that also incorporates factors influencing adherence is essential for optimal follow-up of pregnant individuals using antidepressants. However, no validated self-report scale currently exists to assess antidepressant adherence during pregnancy specifically.

### 1.1 Aims

This study aimed to develop a comprehensive, self-report psychometric scale for measuring adherence to antidepressants during pregnancy. It also sought to assess the scale's psychometric properties, i.e. reliability and construct validity, in a pregnant population using antidepressants in Norway.

## 2 Methods and materials

### 2.1 Study design and data collection

This cross-sectional study was conducted in Norway using an anonymous electronic questionnaire administered between February 18 and March 18, 2022. Eligibility required only that participants were pregnant or up to one year postpartum and had used antidepressants during pregnancy; no additional comorbidity-related inclusion or exclusion criteria were applied. The participants were recruited through invitations shared via social media platforms, apps and patient organizations. The questionnaire was administered through Nettskjema (https://nettskjema.no), a secure online data collection platform hosted by the University of Oslo. Participants were required to read and agree to the informed consent before accessing the questionnaire, which took approximately 15 min to complete.

The target sample size for this preliminary validation was approximately 100 participants. This target was selected using a liberal rule of thumb of 5:1 participants per scale item for exploratory factor analysis (EFA) and the expectation that this sample would approximate the underlying population structure.

The questionnaire consisted of 51 questions (**Appendix 1**) covering socio-demographic characteristics, pregnancy-related information, mental health assessment using validated scales (Edinburgh Perinatal Depression Scale (EPDS)^16^ and General Anxiety Disorder Scale (GAD-7)^17^), beliefs about medication statements.^18^ It also included the proposed *Medication Adherence Measurement in Pregnancy for Antidepressants* (MAMP-AD) scale.

The STROBE statements were used when writing this paper (**Appendix 2**).

### 2.2 Development of the MAMP-AD scale

The initial MAMP-AD scale included 16 items. These were selected based on: (i) previous qualitative research identifying barriers and facilitators to antidepressant use during pregnancy,^13^^,^^14^ (ii) studies on medication beliefs and risk perception in pregnancy,^18^, ^19^, ^20^, ^21^ and (iii) a review of existing medication adherence scales.^15^ The first nine items assessed the frequency of non-adherent behaviors across different scenarios using a five-point Likert scale (scoring from 0 to 4). The remaining seven items focused on whether the antidepressant was taken exactly as prescribed and explored potential predictors of non-adherence. These were scored on a three-point scale (“no” = 0, “uncertain” = 1, “yes” = 2). The total score ranged from 0 to 50, with higher scores indicating better adherence. The pregnancy-wide recall period was selected because medication use and adherence patterns are influenced by evolving symptoms and shifting benefit-risk considerations across the entire pregnancy, and a shorter fixed window could fail to capture these meaningful fluctuations in behavior compared to outside the setting of pregnancy.

A detailed overview of the 16 items and their relevance is provided in **Table S2**.

The questionnaire was piloted for face validity by five pregnant individuals and new mothers to ensure comprehensibility. Minor layout and technical improvements were made based on their feedback. The team working on the scale development included researchers and health care professionals with backgrounds in pharmacy, social sciences, as well as expertise in pharmacotherapy during pregnancy. This ensured that the instrument comprehensively covered the key aspects of the construct being measured.

### 2.3 Validated scales in the study

The questionnaire incorporated several validated scales to assess mental health symptoms and beliefs about medication use during pregnancy.

The EPDS^16^ is a 10-item self-report scale that measures depression and anxiety symptoms over the past seven days. Each item is scored from 0 to 3, with a maximum score of 30. A score above 13 is generally used to indicate clinically relevant depressive symptoms.^22^

The GAD-7 scale measures anxiety symptoms over the past 14 days. It consists of seven items, each scored from 0 to 3. Total scores range from 0 to 21 and are categorized as minimal (0–4), mild (5–9), moderate (10–14), or severe (15–21). A score of 10 or higher is commonly used as the cut-off for clinically relevant anxiety in screening contexts.^17^

The Pregnant Women's Beliefs about Medication Scale^18^ includes six statements specifically designed to measure attitudes towards medication use during pregnancy. Participants responded using the following options: strongly agree, agree, uncertain, disagree, and strongly disagree. Two statements reflect positive beliefs about medication in pregnancy, such as saving unborn children or treating maternal illnesses. The remaining four statements reflect concerns, including potential harm to the fetus, reluctance to take medication even when ill, a raised threshold for medication use, and perceptions of prescribers overprescribing.

### 2.4 Statistical analysis

Development and psychometric evaluation of the MAMP-AD scale were conducted in accordance with established guidelines for scale development and validation.^23^^,^^24^

*Exploratory factor analysis*^25^: An EFA using principal-factor extraction on the estimated correlation matrix was conducted to identify the underlying dimensions (i.e., latent factors) of the MAMP-AD scale. In brief, the EFA models the shared variance among items, capturing the variance explained by common underlying factors rather than item-specific or random error. Pearson correlation matrix was used as a pragmatic solution because polychoric correlations require larger samples and sufficient variability across ordinal response categories, which were not met in the dataset. Additionally, the consistency of the factor solution was assessed by conducting an EFA using a Spearman correlation matrix. The Kaiser-Meyer-Olkin Measure of Sampling Adequacy and Bartlett's Test of Sphericity^26^ were used to verify that the data met the necessary assumptions for EFA. Factors with eigenvalues greater than 1 were retained, in accordance with Kaiser's criterion.^27^ To improve interpretability, the retained factors were rotated using varimax rotation, an orthogonal method that assumes factors are uncorrelated. This statistical technique maximized the variance of item loadings within each factor, making it easier to identify which items were most strongly associated with each factor.^28^ Items were assigned to specific factors based on their factor loadings. The factor loading quantifies the strength and direction of the relationship between an item and a given factor. An item was considered to load onto a factor if its loading was ≥0.4 and clearly exceeded its loadings on other factors. This threshold ensured that items were grouped according to their strongest association with one underlying factor. This, in turn, facilitated defining and labeling of each factor based on the thematic content of its items.

*Reliability Analysis:* Cronbach's α was calculated to assess the internal consistency of the MAMP-AD scale, indicating how closely related the items are as a group. A Cronbach's α coefficient of 0.70 or higher is generally considered acceptable, while very high values (≥0.90) may indicate excessive items.^29^ Separate Cronbach's α coefficients were also computed for each factor of the scale.

*Construct Validity Analysis:* The construct validity of the final MAMP-AD scale was assessed through a series of pre-specified hypotheses, informed by existing literature. We hypothesized that MAMP-AD scores would be:

1) Negatively associated with higher severity of depressive and anxiety symptoms, as measured by EPDS and GAD-7, consistent with prior research^30^^,^^31^;
2) Positively associated with the number of days in the past week that participants reported taking antidepressants exactly as prescribed. This measure served as a simple indicator of adherence over the past week;
3) Positively associated with the perceived benefit of antidepressant treatment, as measured on a scale from 0 (“not at all effective”) to 10 (“very effective”);
4) Higher among participants who agreed with the positive statements from the Pregnant Women's Beliefs About Medication Scale. Conversely, scores would be lower among those who agreed with the negative statements.^32^
5) Higher among participants who believed that continuing antidepressant treatment during pregnancy was the best option. Scores were expected to be lower among those who favored discontinuation.

To examine our first hypothesis, depression (EPDS ≥13) and anxiety (GAD-7 ≥ 10) scores were dichotomized, and analyses were restricted to pregnant participants using antidepressants at the time of questionnaire completion. For the second hypothesis, self-reported adherence over the past week was treated as a binary variable, with ≥6 days considered as being adherent (i.e., >80% adherence), a threshold commonly used in adherence research.^33^ For the third hypothesis, we categorized the perceived benefit of treatment as a binary outcome. High perceived benefit was defined as scores in the upper quartile (above 7.5 on the scale from 0 to 10). In all three hypotheses, MAMP-AD scores were used as continuous predictors in logistic regression models. Results were expressed as odds ratios (ORs) with 95% confidence intervals (CIs). Although the study was cross-sectional and temporality cannot be assessed, we expect that recent depression and anxiety severity, self-reported adherence, and perceived treatment benefit primarily reflect participants' overall antidepressant adherence across pregnancy. This overall adherence is measured by the MAMP-AD scale. Hypotheses four and five were examined using linear regression, with results presented as mean differences (MD) in the MAMP-AD score, along with 95% CIs.

All statistical analyses were conducted using Stata SE Version 18.

## 3 Results

### 3.1 Study population characteristics

**Fig. 1:**
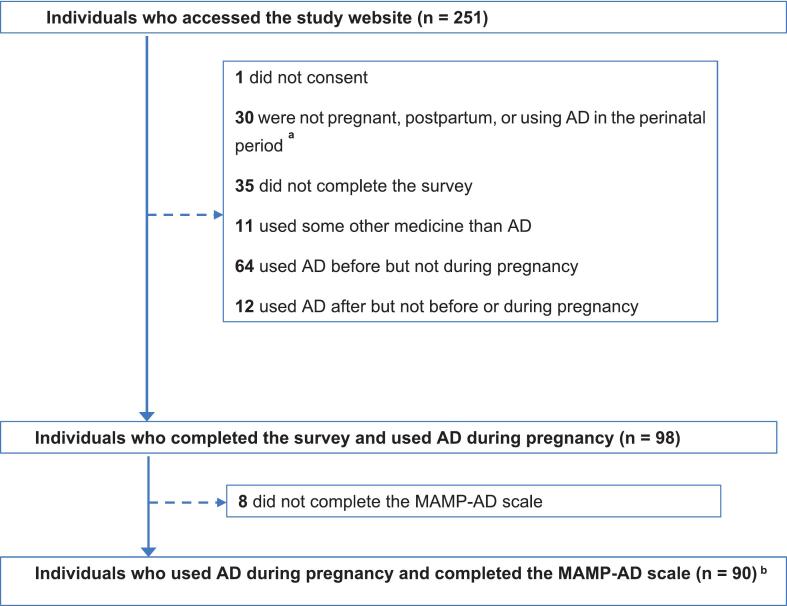
Flow chart of study participants. **Abbreviations and notations: AD** – antidepressants; **MAMP-AD** - Medication Adherence Measurement in Pregnancy for Antidepressants; ^**a**^ Individuals who used AD a year before, during, and a year after pregnancy were invited to fill out the survey; ^**b**^ 89 participants completed all 16 questions from the MAMP-AD scale, while one participant missed the last 16th question, which was then assigned the mean value of the rest of the sample.

Fig. 1 shows the inclusion of study participants. The final study population consisted of 90 participants who used antidepressants during pregnancy and completed the MAMP-AD scale; 49 were pregnant and 41 were postpartum at the time of questionnaire completion. Table 1 presents the socio-demographic, lifestyle, and health-related characteristics of the study participants. The participants' mean age was 31 years (standard deviation SD: 4), and almost all (96%) had a partner. Approximately 70% had a high level of education, and 73% were employed, with nearly one in five working in the healthcare sector. Slightly over 30% of pregnancies were unplanned, and very few (*n* < 5) reported using alcohol or cigarettes during pregnancy. Of the 90 study participants, 83 initiated antidepressants before pregnancy, while seven participants started antidepressants during pregnancy. The most frequently used antidepressant during pregnancy was escitalopram (46%), followed by sertraline (27%) **(****Table S3****).** The mean scores on the EPDS scale were 9.7 (SD: 4.9) for pregnant and 7.6 (SD: 4.8) for postpartum participants. Thirty-one percent of pregnant participants and 24% of postpartum participants scored above the threshold of 13 points, indicating clinically relevant depressive symptoms. On the GAD-7 scale, 29% of pregnant and 15% of postpartum participants scored above 10 points, indicating moderate-severe anxiety symptoms **(****Table S4****).**

**Table 1:** Socio-demographic, lifestyle, and health-related characteristics of the study population, *n* = 90. Numbers are presented as n (%) unless otherwise specified.

Characteristics	Number (%)
Status	
Given birth in the last 12 months	41 (45.6)
Pregnant*	49 (54.4)
*1st trimester*	*13 (26.5)*
*2nd trimester*	*17 (34.7)*
*3rd trimester*	*18 (36.7)*
Maternal age, Mean (sd)	31.4 (4.2)
Parous	27 (30.0)
Married / cohabiting	86 (95.6)

Level of education	
*University / College*	65 (72.2)
Other Education**	24 (26.7)
Norwegian native language	85 (94.4)

Occupation	
*Healthcare worker*	*16 (17.8)*
*Employed but not in healthcare*	*50 (55.6)*
*Other*	*24 (26.7)*
Planned pregnancy	62 (68.9)
No alcohol during pregnancy	86 (95.6)
No smoking during pregnancy	86 (95.6)

Mental health conditions before/during pregnancy	
*Depression*	*20 (22.2)*
*Anxiety*	*6 (6.7)*
*Comorbid depression/anxiety*	*60 (66.7)*
*Other mental disorders*	*<5*
Psychotherapy during pregnancy***	39 (43.3)
Antidepressant use before pregnancy	83 (92.2)

**Abbreviations and notations: n** - sample size; **sd** - standard deviation; (*) One participant did not specify the trimester. (**) One participant did not specify her education. (***) Four participants did not indicate whether they underwent psychotherapy during pregnancy.

### 3.2 Development of the MAMP-AD scale

#### 3.2.1 Exploratory factor analysis

In the initial EFA analysis of the 16-item version of the MAMP-AD scale, the rotated factor matrix revealed four factors. However, Factor 3 and Factor 4 included only three items, and *Item 15* exhibited low loadings on all factors **(****Table S5****)**. Analyses of the distribution of the items **(****Table S6****)** revealed high left skewness on all items, except for *Item 15*. The poor performance of the original *Item 15*, shown by low factor loadings across all factors and high uniqueness, may be due to its strong wording and negative phrasing. The item emphasized the harmful consequences of untreated illness, which may have influenced how participants responded. Its exclusion led to a slight improvement in internal consistency, supporting its removal from the final scale. After reanalysis, three conceptually coherent factors emerged, named based on the content of their loaded items:

• Factor 1: Minimizing Exposure (Items 2, 3, 4, 11, 16)
• Factor 2: Barriers to Antidepressant Use (Items 1, 5, 6, 7, 8, 9) and
• Factor 3: Benefits of Antidepressant Use (Items 10, 12, 13, 14) **(**Table 2**,** Table 3**).**

**Table 2:** Exploratory factor analysis* - Rotated factor loading matrix** and unique variances***.

Variable	Factor 1	Factor 2	Factor 3	Uniqueness
ITEM 1		0.45		0.68
ITEM 2	0.84			0.27
ITEM 3	0.82			0.24
ITEM 4	0.57		0.43	0.43
ITEM 5		0.56		0.69
ITEM 6		0.59		0.65
ITEM 7		0.58		0.62
ITEM 8		0.60		0.56
ITEM 9		0.52		0.63
ITEM 10	0.51		0.69	0.25
ITEM 11	0.83			0.26
ITEM 12			0.66	0.54
ITEM 13			0.46	0.73
ITEM 14			0.45	0.72
ITEM 16	0.45			0.68

Notations: (*) Exploratory factor analysis conducted without item 15, which previously exhibited low factor loadings across all factors and was therefore excluded. (**) Loadings lower than 0.4 are blanked. (***) Item uniqueness represents each item's specific and error variance, calculated as 1 minus the total variance explained by all factors.

**Table 3:** Exploratory factor analysis - Three-factor solution and item content.

Minimizing exposure Factor 1		Barriers to antidepressant use Factor 2		Benefits of antidepressant use Factor 3	
Item	Name	Item	Name	Item	Name
2	Skip or stop when feeling better	1	Forgetfulness	10	Precisely as doctor has prescribed
3	Reducing dose when feeling better	5	Do not improve symptoms	12	Benefits greater than disadvantages
4	Unsafe for child	6	Bluntness	13	Better care for the child
11	Take only when symptoms get worse	7	Financial/practical reasons	14	Preventing relapse
16	Trust in doctor's judgment	8	Prioritize other medications		
		9	Side effects

These three factors explained 81.7% of the total variance in the MAMP-AD scale. The final MAMP-AD scale consisted of 15 items, scoring from 0 to 48, with higher scores indicating higher adherence.

EFA using a Spearman correlation matrix indicated exclusion of items 15 and 1, but produced a factor solution broadly similar to the Pearson-based EFA **(****Table S7****).**

#### 3.2.2 Reliability

Cronbach's α was 0.79 for the 16-item version and 0.80 for the 15-item version of the MAMP-AD scale, indicating acceptable internal consistency and reliability. The Cronbach's α for Factor 1 was 0.84, 0.71 for Factor 2, and 0.69 for Factor 3.

#### 3.2.3 Construct validity

Each one-point increase in the 15-item MAMP-AD scale score was associated with a 26% lower odds of having clinically relevant depressive symptoms (EPDS score ≥ 13; OR: 0.74, 95% CI: 0.56, 0.99) **(*****Fig. S1A******)*.** No association was found between MAMP-AD score and anxiety symptoms (GAD-7 ≥ 10; OR: 1.13, 95% CI: 0.88, 1.44).

Each one-point increase in the MAMP-AD scale score was associated with 32% higher odds of self-reporting being adherent to antidepressants in pregnancy at least six of the past seven days (OR: 1.32; 95% CI: 1.13, 1.54) **(*****Fig. S1B******).*** Additionally, each one-point increase in the MAMP-AD score was associated with 26% higher odds of perceiving antidepressants as highly beneficial (score > 7.5 out of 10) (OR: 1.26, 95% CI: 1.10, 1.43) **(*****Fig. S1C******).***

Women's beliefs about medication showed little impact on MAMP-AD scores. Only belief number 6, “*Doctors prescribe too many medicines to pregnant women*”, was associated with the MAMP-AD score. Participants agreeing with this statement scored on average seven points lower on the MAMP-AD compared to those who disagreed (MD: -7.0; 95% CI: −11.8, −2.2). This belief explained 11% of the variation in the MAMP-AD score **(**Table 4**).**

**Table 4:** Construct validity - Associations between statements from Pregnant Women's Beliefs about medication* and the MAMP-AD scale score**.

Statements from Beliefs about Medication in Pregnancy	n	MAMP-AD scale score	Mean difference on the MAMP-AD scale score (95% CI)	R^2^
1: All medicines can be harmful to the fetus.				
Agree	7	42.1	−2.9 (−6.6, 0.9)	0.04
Uncertain	11	43.4	−1.6 (−4.7, 1.4)	
Disagree	72	45.0	Ref.	

2: Even if I'm ill, I believe it's better for the fetus that I refrain from using medicines during pregnancy.				
Agree	9	43.3	−1.9 (−5.2, 1.5)	0.05
Uncertain	16	42.8	−2.5 (−5.1, 0.2)	
Disagree	64	45.2	Ref.	

3: I have a higher threshold for using medicines when pregnant than when I'm not pregnant.				
Agree	78	44.7	0.2 (−3.0, 3.4)	0.02
Uncertain	2	40.0	−4.5 (−11.9, 2.9)	
Disagree	10	44.5	Ref.	

4: Thanks to treatment with medicines during pregnancy, the lives of many unborn children are saved each year.				
Agree	50	44.3	1.8 (−3.2, 6.7)	0.02
Uncertain	36	45.2	2.7 (−2.3, 7.8)	
Disagree	4	42.5	Ref.	

5: It is better for the fetus that I use medicines and get well than to have an untreated illness during pregnancy.				
Agree	66	44.4	−0.2 (−4.0, 3.6)	0.01
Uncertain	17	45.3	0.7 (−3.6, 5.0)	
Disagree	7	44.6	Ref.	

6: Doctors prescribe too many medicines to pregnant women.				
Agree	**4**	**37.5**	**−7.0 (−11.8, −2.2)**	**0.11**
Uncertain	54	45.1	0.6 (−1.4, 2.6)	
Disagree	32	44.5	Ref.	

**Abbreviations and notations: n** - sample size; **MAMP-AD** - Medication Adherence Measurement in Pregnancy for Antidepressants; **CI** - Confidence interval; **R**^**2**^ - Coefficient of determination; (*)The statements were trichotomized (agree, disagree, or uncertain) in the analyses (**) The score on the 15-item MAMP-AD scale ranges from 0 to 48.

**Significant results are bolded.**

Furthermore, participants who believed that continuing antidepressant treatment during pregnancy was the best option scored on average 4.6 points higher on the MAMP-AD scale compared to those who preferred discontinuing (MD: 4.6; 95% CI: 1.9, 7.4) **(**Table 5**).**

**Table 5:** Construct validity - Association between a specific statement about antidepressant use in pregnancy and the MAMP-AD score*, *n* = 89**.

What do you think is/was best for you to do with antidepressant treatment when you became pregnant?				
	n	MAMP-AD score	Mean difference on the MAMP-AD scale (95% CI)	R^2^
• Continue treatment with the same antidepressant(s)	39	46.6	**4.6 (1.9, 7.4)**	0.2
• Switch to another antidepressant	5	43.4	1.5 (−3.2, 6.1)	
• Reduce the dose of the antidepressant	23	43.3	1.3 (−1.7, 4.3)	
• Other	7	44.1	2.2 (−1.9, 6.3)	
• Discontinue use of the antidepressant	15	41.9	Ref.	

**Abbreviations and notations: n** - sample size; **MAMP-AD** - Medication Adherence Measurement in Pregnancy for Antidepressants; **CI** - Confidence interval; **R**^**2**^ - Coefficient of determination; (*) The score on the 15-item MAMP-AD scale ranges from 0 to 48. **One participant did not fill out this question. **Significant results are bolded.**

### 3.3 MAMP-AD scale scores

The mean score on the 15-item MAMP-AD scale was 44.6. It remained largely consistent across different assessment times (i.e., participants in different trimesters of pregnancy or in the postpartum period, when they retrospectively reported on their antidepressant use during pregnancy). Median scores were 46 for pregnant and 47 for those answering postpartum **(****Table S4****).**

## 4 Discussion

In this study, the final 15-item MAMP-AD scale demonstrated a clear three factor structure, i.e., **Minimizing exposure, Barriers to antidepressant use,** and **Benefits of antidepressant use,** and showed acceptable reliability (Cronbach's α = 0.80). As hypothesized, antidepressant adherence scores as measured by the MAMP-AD were negatively associated with depressive symptom severity and positively associated with self-reported adherence over the past week and perceived treatment benefits. These findings provide preliminary evidence of construct validity of the MAMP-AD scale.

Following the removal of one item (original *Item 15*), the refined factor structure aligned with established determinants of antidepressant adherence and broader patterns of medication adherence during pregnancy. **Factor 1 “Minimizing exposure”** (Items 2, 3, 4, 11, 16) reflected decisions to reduce antidepressant use based on symptoms and the perception that it is safer for the child. Prior studies have shown that self-rated severity of illness^34^ and safety concerns during pregnancy^11^^,^^35^^,^^36^ play an important role in medication adherence in pregnancy. Moreover, it is documented that pregnant individuals perceive antidepressants as a high-risk medication.^21^ **Factor 2 “Barriers to antidepressants use”** (Items 1, 5, 6, 7, 8, 9) captured important patient-related barriers such as forgetfulness, medication-related barriers like side effects, and practical barriers such as financial issues. These barriers are known to influence general adherence.^12^^,^^37^ They may be further accentuated during pregnancy, which can make decisions about antidepressant use more difficult.^36^ **Factor 3 “Benefits of antidepressant use”** (Items 10, 12, 13, 14) reflected perceived usefulness/benefit of the antidepressant treatment. This perception has also been identified in earlier research as an important predictor of adherence.^13^

Cross-loading refers to an item loading significantly onto more than one factor, suggesting it may be conceptually related to multiple underlying dimensions. Items 4 and 10 exhibited notable cross-loadings, with Item 4 showing a primary loading on Factor 1 and a secondary loading on Factor 3, whereas Item 10 showed the opposite pattern. Item 10 differs content-wise from all other items, as it asks whether antidepressants were used precisely as prescribed, without addressing motivation or underlying reasons. This distinction may explain the observed cross-loading.

Several scale items showed high uniqueness (i.e. values above 0.6). This indicates that the three factors explain less than 40% of the variance in those items, reflecting low communalities. Multiple issues likely contributed to these low loadings and communalities. First, many items showed left-skewed distributions and limited response variability in our study population. These characteristics can reduce inter-item covariance and weaken factor loadings. Second, items with only three ordinal response levels may have violated the assumption of linearity, potentially leading to underestimation of factor loadings.^38^ The combination of low communalities and the relatively small sample size (*n* = 90) raises concerns about the robustness of factor determination, particularly whether each factor is adequately represented by a sufficient number of items.^39^^,^^40^ Although the three factors were represented by five, six, and four items respectively, two items exhibited notable cross-loadings. This suggests conceptual overlap and raises questions about the clarity of the factor structure. These findings underscore the need for further examination of the scale's dimensionality in larger and more diverse samples to ensure the stability and interpretability of the factor structure.^39^

Nonetheless, the three distinct factors align with existing adherence literature,^12^^,^^36^ and the 15-item MAMP-AD scale demonstrated acceptable reliability and satisfactory construct validity. With these promising preliminary findings, the MAMP-AD scale has the potential to contribute to both clinical practice and research in several ways. Firstly, analyzing adherence patterns in larger and more diverse populations could help identify subgroups at higher risk of non-adherence, informing tailored interventions and supporting timely implementation of broader adherence strategies. Secondly, the scale may facilitate communication between patients and healthcare providers by offering a structured tool to discuss challenges and motivations related to medication use, thereby facilitating more personalized and effective treatment plans. Thirdly, beyond clinical use, the scale may serve as a valuable psychometric tool in research. For example, it can be used in longitudinal studies examining short- and long-term effects of antidepressant use during pregnancy, in studies exploring the relationship between adherence and pregnancy outcomes, and in comparative studies assessing interventions to improve adherence.

Previous studies on antidepressant adherence in the pregnant population have been limited by the lack of validated, pregnancy-specific assessment tools, leading to inconsistent findings and challenges in interpreting results. A validated scale such as the MAMP-AD could enable more rigorous, reliable, and comparable future studies by enabling accurate measurement of antidepressant adherence and its determinants. Given the importance of antidepressant adherence in managing peripartum depression and the serious consequences of low adherence for both the mother and child,^41^ a validated adherence scale for pregnant populations is greatly needed. Such a scale could enhance clinical practice, inform guidelines, and advance future research in this field.

### 4.1 Strengths and limitations

A main strength of this study is the use of anonymous data collection, which likely reduced social desirability bias. This is an important advantage given the stigma often associated with mental illness and antidepressant use.^34^ Additionally, the study followed a structured approach to scale development.^23^^,^^24^ We also used several validated scales to assess reliability and validity,^16^, ^17^, ^18^ enhancing the robustness of the results.

However, several limitations should be acknowledged. Firstly, the small sample size limits statistical power. In small samples, the inclusion of extreme values may disproportionately influence model estimates. Moreover, the sample size constrained our ability to fully evaluate the scale's performance across pregnant versus postpartum participants. Secondly, the inclusion of participants with varying recall periods and diverse experiences may introduce information bias, potentially affecting the reliability of adherence estimates in subpopulations. Additionally, because adherence was assessed through self-report rather than objective measures such as prescription refills or pill counts, our findings may be subject to information bias, highlighting the need for future studies to incorporate multiple complementary adherence assessment methods. Lastly, the recruitment strategy, primarily through health and pregnancy-related social media pages and apps, may have resulted in a sample comprising primarily well-informed and treatment-engaged participants. This could limit the generalizability of findings, as the experiences of less engaged or more vulnerable populations may not be adequately represented.

### 4.2 Future research

Further investigation of the underlying dimensions of the MAMP-AD scale in larger and more diverse samples is needed to help refine item inclusion and ensure that the scale is valid, reliable, and sensitive to real differences in adherence behavior. To better account for the ordinal nature of the data, alternative approaches to the correlation matrix in EFA—such as the use of polychoric correlations—could be employed.^38^^,^^42^ Additionally, scale scoring strategies (i.e., methods for calculating and interpreting total and subscale scores) should be further explored. This will help optimize the interpretability and clinical utility of the scale. Future work should also explore pathways for integrating the MAMP-AD into routine clinical workflows and digital platforms, including pregnancy apps and electronic health systems, to support scalable monitoring and personalized follow-up. Furthermore, the scale's criterion validity should be assessed against established adherence measures and objective indicators, and its test–retest reliability evaluated. A larger sample would also allow for stratified analyses to adjust for additional relevant variables, such as antidepressant type and pregnancy trimester. Larger samples will also make it possible to examine how MAMP-AD scores differ between individuals who initiated antidepressants during pregnancy and those already on treatment before conception, allowing evaluation of the scale across clinically distinct adherence scenarios. This would strengthen the robustness of hypothesis testing in future construct validity analysis.

## 5 Conclusion

The newly developed MAMP-AD scale showed promising reliability and validity as a self-reported measure of antidepressant adherence during pregnancy. It offers a structured tool for both research and clinical settings, enabling the identification of adherence barriers and supporting more targeted interventions. Future studies in larger and more diverse populations are needed to further evaluate and confirm the scale's psychometric properties.

## Ethical perspectives

As this was an anonymous survey, the study was exempt from formal ethical review and did not require approval from the Regional Committees for Medical and Health Research Ethics (REK). All participants gave informed consent before participating in the study. This study was conducted as part of a master's thesis at the Department of Pharmacy at the University of Oslo under the name “My medicines and me-study” (Norwegian: “Mine medisiner og jeg-studie”: Mine medisiner og jeg-studien (avsluttet) - Farmasøytisk institutt (uio.no)).

## CRediT authorship contribution statement

**Milica Zugic:** Writing – review & editing, Writing – original draft, Methodology, Formal analysis. **Natasa Pejic:** Writing – review & editing, Methodology, Formal analysis, Data curation, Conceptualization. **Saeed Hayati:** Writing – review & editing, Methodology. **Marleen M.H.J. van Gelder:** Writing – review & editing, Methodology. **Hedvig Nordeng:** Writing – review & editing, Writing – original draft, Supervision, Project administration, Methodology, Data curation, Conceptualization.

## Declaration of generative AI and AI-assisted technologies in the writing process

During the preparation of this work, the author(s) used GPT UIO (gpt.uio.no) to improve the readability and language. After using this tool, the author(s) reviewed and edited the content as needed and take(s) full responsibility for the content of the published article.

## Declaration of competing interest

The authors declare that they have no known competing financial interests or personal relationships that could have appeared to influence the work reported in this paper.

## Appendix A Supplementary data

Supplementary material Image 1
