# Supplementary material for: Development of a novel adherence scale for antidepressants in pregnancy: Results from a cross-sectional study
Source: Explor Res Clin Soc Pharm. 2026 Jan 12;21:100704. doi: 10.1016/j.rcsop.2026.100704 (PMC12905790; doi:10.1016/j.rcsop.2026.100704)
Supplement: Supplementary file 1 — Supplementary material [file mmc1.docx]

**Development of a novel adherence scale for antidepressants in pregnancy: results from a cross-sectional study**

Supplementary material

[Appendix 1: Questionnaire in English 3](#_Toc216646500)

[Appendix 2: STROBE Statement—checklist of items that should be included in reports of observational studies 19](#_Toc216646501)

[Table S1: Overview of studies on adherence to antidepressants in pregnancy 22](#_Toc216646502)

[Table S2: The 16 MAMP-AD* scale items with their scientific rationale 24](#_Toc216646503)

[Table S3: Antidepressants used in the study population (n=90) 27](#_Toc216646504)

[Table S4: Distribution of EPDS, GAD-7 and MAMP-AD scores during pregnancy by trimester and postpartum 28](#_Toc216646505)

[Table S5: Exploratory factor analysis - Rotated factor loading matrix* and unique variances** for all 16 MAMP-AD scale items, providing the rational for excluding item 15 (low factor loading, high uniqueness) 29](#_Toc216646506)

[Table S6: Summary statistics: The distributional properties of individual items in the MAMP-AD scale. Data from the exploratory factor analysis (EFA). 30](#_Toc216646507)

[Table S7: Exploratory factor analysis based on Spearman correlations - rotated factor loading matrix* and unique variances** for 14 MAMP‑AD items*** 31](#_Toc216646508)

[Figure S1: Construct validity: association between antidepressant adherence (MAMP-AD score*) and depressive symptoms (EDPS) (A), number of days in a week antidepressants were taken exactly as prescribed (B) and perceived antidepressant benefit (C) 32](#_Toc216646509)

[References 33](#_Toc216646510)

## Appendix 1: Questionnaire in English

***INFORMATION ABOUT YOURSELF***

1. **Please specify your current status**.

🞎 I am pregnant

🞎 I have given birth in the last 12 months

1. (If ticked “I am pregnant” in Q1): **In which pregnancy week are you?**

From 1 to 44

1. (If ticked “I have given birth in the past 12 months” in Q1) **How old is your child?**

🞎 Less than one month

🞎 1-3 months

🞎 4-6 months

🞎 7-9 months

🞎 10-12 months

1. **Have you been pregnant before? (This also applies to pregnancy that ended in abortion, miscarriage, or fetal death)**

🞎 Yes 🞎 No

1. **a)** (If ticked “I am pregnant” in Q1): **How many children do you have from before,** **excluding your current pregnancy?**

🞎 None 🞎 1 🞎 2 🞎 more than 2

**b)** (If ticked “I have given birth in the last 12 months” in Q1) **How many children do you have from before,** **excluding your last child?**

🞎 None 🞎 1 🞎 2 🞎 more than 2

1. **What is your marital status?**

🞎 Married 🞎 Cohabitant 🞎 Single 🞎 Divorced/Separated 🞎 Widow

🞎 Other/Please specify: ______________________________

1. **What is the highest education you have completed?**

🞎 Primary school (10 years of education)

🞎 High school (11-13 years of education)

🞎 University / college

🞎 Other/Please specify: _________________________________

1. **What is your age? (In years): _________________________**
2. **Is Norwegian your (one of your) mother tongue(s)?** 🞎 Yes 🞎 No

***YOUR CURRENT OR MOST RECENT PREGNANCY***

1. **Was your pregnancy planned?**

🞎 Yes 🞎 No, but it was not completely unexpected 🞎 No, it was not planned

1. **Did you drink any alcohol after finding out that you were pregnant?**

🞎 No 🞎 Once or twice 🞎 Several times, but not daily 🞎 Daily  🞎 Cannot remember

1. **Did you smoke after finding out you were pregnant?**

🞎 No 🞎 Once or twice 🞎 Several times, but not daily 🞎 Daily 🞎 Cannot remember

1. **What was your work situation when you became pregnant?**

🞎 Student

🞎 Homemaker

🞎 Health care personnel (i.e., physician, nurse, or pharmacist)

🞎 Employed in another sector

🞎 Jobseeker or laid off

🞎 On a sick leave

🞎 None of the above, specify: _________________________________________________

1. **Who followed you up during your current or most recent pregnancy? You may tick more than one option**.

🞎 Midwife at the health station

🞎 My GP

🞎 Specialist psychologist (at the DPS or private)

🞎 Specialist psychiatrist (at the DPS or private)

🞎 Specialist team at the hospital

🞎 Specialist team at the health station

🞎 I was not followed up

🞎 Other, specify: ___________________________

**HOW YOU ARE DOING AND YOUR MEDICATION USE**

The following questions are about your well-being and your use of medication in the period around pregnancy.

1. **a) Have you or have you had any of the following mental illnesses or health problems in the period around your pregnancy? If yes, check the box when you have experienced the illnesses. Please choose the alternatives that apply to you**.

|  | Before pregnancy | During the pregnancy | After pregnancy |
| --- | --- | --- | --- |
| 🞎 Depression |  |  |  |
| 🞎 Anxiety |  |  |  |
| 🞎 Obsessive-Compulsive Disorder (OCD) |  |  |  |
| 🞎 Bipolar disorder |  |  |  |
| 🞎 Eating disorder |  |  |  |
| 🞎 Other mental problem/illness |  |  |  |

**b) If you checked for «other health problem/illness» in the previous question, please specify which health problem/illness you had and for how long?** _______________________________________________________________________________________________________________________________________________________________________________________________________________________________________________________________

1. **a)** (If ticked “I am pregnant” in Q1): **Have you received or receive now psychological treatment for your mental health problem(s)/illness(es) during your current pregnancy?**

🞎 Yes, I receive or have received psychological treatment during the pregnancy

🞎 No, I do not receive or have not received any psychological treatment during the pregnancy

1. (If ticked “I have given birth in the last 12 months” in Q1): **Have you received psychological treatment for your mental health problem(s)/illness(es) during your most recent pregnancy?**

🞎 Yes, I have received psychological treatment during the pregnancy

🞎 No, I have not received any psychological treatment during the pregnancy

1. **During the past month: have you often been bothered by feelings of sadness, depression or hopelessness?**

🞎 Yes 🞎 No

1. **During the past month: have you often been bothered by having less interest in things or less pleasure in doing things?**

🞎 Yes 🞎 No

**The following 10 questions are about how you have been doing it for the last 7 days. There are no right or wrong answers. We are only interested in your personal views. (Tick only one box per question)**

**[Questions 19-28:** In this section of the questionnaire, the **Edinburgh Perinatal Depression Scale (EPDS)** was presented (*Cox JL, Holden JM, Sagovsky R. Detection of postnatal depression. Development of the 10-item Edinburgh Postnatal Depression Scale. Br J Psychiatry. 1987;150(6):782-6. https://doi.org/10.1192/bjp.150.6.782*).**]**

1. **Over the last 2 weeks, how often have you been bothered by the following problems? (You may only tick once per line)**

|  | Not at all | Several days | More than half of the days | Nearly every day |
| --- | --- | --- | --- | --- |
| 1. **Feeling nervous, anxious or on edge** | **□** | **□** | **□** | **□** |
| 1. **Not being able to stop or control worrying** | **□** | **□** | **□** | **□** |
| 1. **Worrying too much about different things** | **□** | **□** | **□** | **□** |
| 1. **Trouble relaxing** | **□** | **□** | **□** | **□** |
| 1. **Being so restless that it is hard to sit still** | **□** | **□** | **□** | **□** |
| 1. **Becoming easily annoyed or irritable** | **□** | **□** | **□** | **□** |
| 1. **Feeling afraid as if something awful might happen.** | **□** | **□** | **□** | **□** |
| 1. **If you checked off any problems, how difficult have these problems made it for you to do your work, take care of things at home, or get along with other people?**   **□** Not difficult at all **□** Somewhat difficult **□** Very difficult **□** Extremely difficult | | | | |

**[**The first seven subquestions under Question 29 are part of the **Generalized Anxiety Disorder 7-item (GAD-7) screening scale**. This scale was developed by Drs. Robert L. Spitzer, Janet B.W. Williams, Kurt Kroenke and colleagues, with an educational grant from Pfizer Inc (*Spitzer RL, Kroenke K, Williams JBW, Löwe B. A Brief Measure for Assessing Generalized Anxiety Disorder: The GAD-7. Arch Intern Med. 2006;166(10):1092-7. https://doi.org/10.1001/archinte.166.10.1092*). Translated version of the GAD-7 scale can be found on the official website at [*https://www.phqscreeners.com/*](https://www.phqscreeners.com/)**]**

(If ticked “I am pregnant” in Q1): **The following questions are about the occurrence of nausea during your pregnancy.**

1. (If ticked “I am pregnant” in Q1): **Circle the answer that suits the best your situation for the last 24 hours:**

| 1. **On average, in a day, for how long do you feel nauseated or sick to your stomach?**   □ > 6 hours □ 4-6 hours □ 2-3 hours □ ≤ 1 hour □ Not at all |
| --- |
| 1. **On average in a day, how many times do you vomit or throw up**   □ ≥ 7 times □ 5-6 times □ 3-4 times □ 1-2 times □ Not at all |
| 1. **On average in a day, how many times have you had retching or dry heaves without bringing anything up?**   □ ≥7 times □ 5-6 times □ 3-4 times □ 1-2 times □ Not at all |

1. (If ticked “I am pregnant” in Q1): **On a scale of 0 to 10 (0= worst possible, 10= as good as you felt before pregnancy), how would you rate your well-being:**

Choose: **0 1 2 3 4 5 6 7 8 9 10**

**The following questions are about the antidepressant medication before, during, and after pregnancy**.

1. **What do you think is most correct if a woman using antidepressants becomes pregnant? It is possible to select several options.**

- The use of antidepressants should be continued during pregnancy when needed
- A woman who is to choose between using antidepressants during pregnancy or not should be given information about the advantages and disadvantages of such treatment - so that she can decide for herself what is best for her
- It is up to the doctor to decide if it is best to continue using antidepressants during pregnancy or not
- The use of antidepressants should be stopped as soon as possible if a woman discovers that she is pregnant because antidepressants can affect the fetus in a negative way
- The use of antidepressants should not be discontinued because it can have significant negative consequences for the woman's mental health, and thus also the child's health
- I have no opinion about this
- Other, specify ___________________________

1. **What do you think is/was best for you to do with antidepressant treatment when you became pregnant?**

- Continue treatment with the same antidepressant(s)
- Switch to another antidepressant
- Discontinue use of the antidepressant
- Reduce the dose of the antidepressant
- No preference
- Other, specify: _________________________________

**The following questions are about your treatment with antidepressant medication.**

1. **Tick the option that is correct for you:**

□ I am using antidepressants in pregnancy now

□ I have used antidepressants during pregnancy

□ None above

□ Do not remember

1. **a) Which antidepressant from the list below are/have you used? Please indicate when you used them.**

|  | More than 6 months before pregnancy | 6 months or less before pregnancy | 1^st^ trimester | 2^nd^ trimester | 3^rd^ trimester | 0 to 12 months after birth |
| --- | --- | --- | --- | --- | --- | --- |
| 🞎 **Fluoxetine** (incl. Fluoxetin Mylan, Fluoxetine Orion, Fontex) |  |  |  |  |  |  |
| 🞎 **Citalopram** (incl. Cipramil Farmagon, Cipramil Lundbeck, Citalopram Sandoz) |  |  |  |  |  |  |
| 🞎 **Escitalopram** (incl. Cipralex Farmagon, Cipralex Lundbeck, Escitalopram Actavis) |  |  |  |  |  |  |
| 🞎 **Paroxetine** (incl. Seroxat, Paroxetin Actavis, Paroxetin Farmagon) |  |  |  |  |  |  |
| 🞎 **Sertraline** (incl. Sertralin HEXAL, Zoloft, Sertraline Accord) |  |  |  |  |  |  |
| 🞎 **Fluvoxamine** (incl. Fevarin Mylan, Fevarin Orifarm) |  |  |  |  |  |  |
| 🞎 **Venlafaxine** (incl. Efexor, Venorion, Venlazid, Venlafaxin Bluefish) |  |  |  |  |  |  |
| 🞎 **Duloxetine** (incl. Cymbalta, Duloxetin Pensa, Duloxetine Mylan) | 󠄿 | 󠄿 | 󠄿 | 󠄿 | 󠄿 | 󠄿 |
| 🞎 **Mirtazapine** (incl. Remeron, Mirtazapin Bluefish) |  |  |  |  |  |  |
| 🞎 **Reboxetine** (incl. Edronax) |  |  |  |  |  |  |
| 🞎 **Mianserin** (incl. Mianserin Mylan, Tolvon) |  |  |  |  |  |  |
| 🞎 **Amitriptyline** (incl. Anafranil, Klomipramin Mylan) |  |  |  |  |  |  |
| 🞎 **Clomipramine** (incl. Anafranil, Klomipramin Mylan) |  |  |  |  |  |  |
| 🞎 **Trimipramine** (incl. Surmontil) |  |  |  |  |  |  |
| 🞎 **Nortriptyline** (incl. Noritren) |  |  |  |  |  |  |
| 🞎 **Doxepine**  (inkl. Sinequan) |  |  |  |  |  |  |
| 🞎 **Vortioksetin**  (ink. Brintelix) |  |  |  |  |  |  |
| 🞎 **I don't remember the name of the medicine** |  |  |  |  |  |  |
| 🞎 **Other antidepressants** |  |  |  |  |  |  |

**b)** **If you checked «other antidepressant(s)» in the previous question, please specify which medicine(s) you used and for how long? ________________________________________________________________________________________________________________________________________________________________**

1. **Was the dose of your prescribed antidepressant changed during pregnancy?**

🞎 Yes, the dose was increased

🞎 Yes, the dose was reduced

🞎 I stopped taking the medication

🞎 No, the dose was the same

🞎 Cannot remember

1. If ticked “I am using antidepressants in pregnancy now” or “I have used antidepressants during pregnancy” in Q34)

**On a scale from 0 (not at all) to 10 (very effective),** **how effective do you think your therapy with antidepressants was/is in treating your illness during pregnancy?**

Choose: 0 1 2 3 4 5 6 7 8 9 10

1. If ticked “I am using antidepressants in pregnancy now” or “I have used antidepressants during pregnancy” in Q34, **Does/did your partner agree that antidepressants are/were the right treatment for you during pregnancy?**

□ Yes, my partner mostly agrees/entirely agrees

□ No, my partner disagrees/strongly disagrees

□ My partner is/was unsure

□ Not applicable

1. **a)** If ticked “I am using antidepressants in pregnancy now” in Q34: **The following questions apply to the period when you are pregnant. Please choose the answer that best reflects** **your use of antidepressants during pregnancy and your opinions on the use of this medicine:**

**DURING PREGNANCY:**

|  | Very often | Often | Sometimes | Seldom | Never |
| --- | --- | --- | --- | --- | --- |
| 1. **How often do you forget to take your antidepressant?** | **□** | **□** | **□** | **□** | **□** |
| 1. **Do you sometimes skip a dose or take a break from your antidepressants when you feel better?** | **□** | **□** | **□** | **□** | **□** |
| 1. **Do you ever reduce the dose of your antidepressant when you feel better? (For example, if you take 1 tablet instead of 2 tablets, or take ½ tablet instead of 1 tablet)** | **□** | **□** | **□** | **□** | **□** |
| 1. **Do you ever use less antidepressants (than prescribed by the doctor) because you think it is safer for your unborn child?** | **□** | **□** | **□** | **□** | **□** |
| 1. **Do you ever avoid taking your antidepressants (as prescribed by the doctor) because they do not improve your symptoms sufficiently?** | **□** | **□** | **□** | **□** | **□** |
| 1. **Do you ever avoid taking your antidepressants (as prescribed by the doctor) because they make you feel emotionally blunt and less like yourself?** | **□** | **□** | **□** | **□** | **□** |
| 1. **Do you sometimes not take your antidepressants (as prescribed by the doctor) because financial or practical reasons limit your access to them?** | **□** | **□** | **□** | **□** | **□** |
| 1. **Do you ever avoid taking your antidepressants (as prescribed by the doctor) because you prioritize taking other medications?** | **□** | **□** | **□** | **□** | **□** |
| 1. **Do you ever avoid taking your antidepressants (as prescribed by the doctor) because they give you too many side effects?** | **□** | **□** | **□** | **□** | **□** |

|  | Yes | Uncertain | No |
| --- | --- | --- | --- |
| 1. **Do you take your antidepressants precisely as the doctor has prescribed?** | **□** | **□** | **□** |
| 1. **Do you take your antidepressants only when you feel your symptoms are getting worse?** | **□** | **□** | **□** |
| 1. **Are the benefits of taking antidepressants greater than the disadvantages for you?** | **□** | **□** | **□** |
| 1. **Do you believe that taking antidepressants will enable you to take better care of your child once it is born?** | **□** | **□** | **□** |
| 1. **Do you believe that taking your antidepressant can prevent disease relapse during pregnancy and/or after birth?** | **□** | **□** | **□** |
| 1. **Do you believe not treating your disorder with antidepressants during pregnancy may harm your unborn child?** | **□** | **□** | **□** |
| 1. **When it comes to your treatment with antidepressants during pregnancy, do you trust the doctor’s judgment?** | **□** | **□** | **□** |

1. If ticked “I have used antidepressants during pregnancy” in Q34, **The following questions apply to the period when you were pregnant. Please choose the answer that best reflects your use of antidepressants during pregnancy and your opinions on the use of this medicine:**

**DURING PREGNANCY:**

|  | Very often | Often | Sometimes | Seldom | Never |
| --- | --- | --- | --- | --- | --- |
| 1. **How often did you forget to take your antidepressants?** | **□** | **□** | **□** | **□** | **□** |
| 1. **Did you sometimes skip a dose or take a break from your antidepressants when you felt better?** | **□** | **□** | **□** | **□** | **□** |
| 1. **Did you ever reduce the dose of your antidepressants when you felt better? (For example, if you took 1 tablet instead of 2 tablets, or ½ tablet instead of 1 tablet)** | **□** | **□** | **□** | **□** | **□** |
| 1. **Did you ever use less antidepressants (than prescribed by the doctor) because you thought it was safer for your unborn child?** | **□** | **□** | **□** | **□** | **□** |
| 1. **Did you ever avoid taking your antidepressants (as prescribed by the doctor) because they did not improve your symptoms sufficiently?** | **□** | **□** | **□** | **□** | **□** |
| 1. **Did you ever avoid taking your antidepressants (as prescribed by the doctor) because they made you feel emotionally blunt and less like yourself?** | **□** | **□** | **□** | **□** | **□** |
| 1. **Did you sometimes not take your antidepressants because financial or practical reasons limited your access to them?** | **□** | **□** | **□** | **□** | **□** |
| 1. **Did you ever avoid taking your antidepressants (as prescribed by the doctor) because you prioritized taking other medications?** | **□** | **□** | **□** | **□** | **□** |
| 1. **Did you ever avoid taking your antidepressants (as prescribed by the doctor) because they gave you too many side effects?** | **□** | **□** | **□** | **□** | **□** |

|  | Yes | Uncertain | No |
| --- | --- | --- | --- |
| 1. **Did you take your antidepressants precisely as the doctor has prescribed?** | **□** | **□** | **□** |
| 1. **Did you take your antidepressants only when you felt your symptoms were getting worse?** | **□** | **□** | **□** |
| 1. **Were the benefits of taking antidepressants greater than the disadvantages for you?** | **□** | **□** | **□** |
| 1. **Did you believe that taking antidepressants will enable you to take better care of your child once it is born?** | **□** | **□** | **□** |
| 1. **Did you believe that taking antidepressants can prevent disease relapse during pregnancy and/or after birth?** | **□** | **□** | **□** |
| 1. **Did you believe not treating your mental disorder during pregnancy may harm your unborn child?** | **□** | **□** | **□** |
| 1. **When it comes to your treatment with antidepressants during pregnancy, did you trust the doctor’s judgment?** | **□** | **□** | **□** |

1. **a)** If ticked “I am using antidepressants in pregnancy now” in Q34: **During the past week, how many days did you take the antidepressant precisely as prescribed by your doctor?**

Number of days: 0 1 2 3 4 5 6 7 Don’t remember

**b)** If ticked “I have used antidepressants during pregnancy” in Q34 **During the last week of taking antidepressants during pregnancy, how many days did you take your medication exactly as prescribed by your doctor?**

Number of days: 0 1 2 3 4 5 6 7 Don’t remember

1. **Do/did you use any tools to remind you to take antidepressants? (You can select several of the options)**

□ Mobile medisin reminder app

□ Sett an alarm

□ Using a pillbox

□ I have a routine that helps me remember to take antidepressants

□ No tools

□ Other, specify _______________________________

1. (If ticked"Yes" in Q32) **What do/did you do if you forget forgot to take a dose of antidepressant (You can select several of the options)**

□ Nothing

□ I never forget/forgot to take a dose of antidepressant

□ I take/took it as soon I remember(ed), unless it is/was near time for my next dose

□ I double(d) the next dose

□ Other, specify _________________________________

1. **Would you like to share some additional comments/experience about your use of antidepressants during pregnancy? Both positive and negative experiences are useful to hear about.** __________________________________________________________________________________________________________________________________________________________________________________________________________________________________________________________________________________________
2. **a) If you are taking or have been taking other medications than antidepressants, please choose relevant medications from the list below and when you were using them. If you have not used any medication other than antidepressants, you can skip the next question.**

|  | 1 year or less before pregnancy | 1^st^ trimester | 2^nd^ trimester | 3^rd^ trimester | After birth |
| --- | --- | --- | --- | --- | --- |
| **Mild painkillers:**  **Paracetamol** (e.g., Panodil, Pinex) | □ | □ | □ | □ | □ |
| **Mild painkillers:**  **NSAID**(e.g.,Ibux, Diclofenac, Voltaren, Ketorolac) | □ | □ | □ | □ | □ |
| **Strong painkillers:**  **Opioid analgesics** (e.g., Paralgin forte, Tramadol) | □ | □ | □ | □ | □ |
| **Mood stabilizers** (e.g., Lamictal, Litium, Lithionit | □ | □ | □ | □ | □ |
| **Antipsychotics** (e.g., Zyprexa, Seroquel) | □ | □ | □ | □ | □ |
| **Anxiolytics** (e.g., Valium, Sobril, Atarax) | □ | □ | □ | □ | □ |
| **Sleeping medications** (e.g., Imovane, Stilnoct, Zolpidem) | □ | □ | □ | □ | □ |
| **Antiepileptics** (e.g., Fenemal, Fenantoin. Rivotril, Tegretol, Trileptal, Zebinix) | □ | □ | □ | □ | □ |
| **Stimulant medications** (e.g. Strattera, Aduvanz, Attentin) | □ | □ | □ | □ | □ |
| **Other medicine(s)** | □ | □ | □ | □ | □ |

**b) If you ticked «other medicine(s)» in the previous question, please specify which medicine(s) you used and for how long?** _______________________________________________________________________________________________________________________________________________________________________________________________________________________________________________________________

***YOUR PERCEPTION OF RISK DURING PREGNANCY***

1. **Please indicate how harmful you think the following items are during pregnancy on a scale from 0 to 10, where 0 corresponds to ‘not harmful’ and 10 to ‘very harmful’.**

|  | **I don’t know** | **0** | **1** | **2** | **3** | **4** | **5** | **6** | **7** | **8** | **9** | **10** |
| --- | --- | --- | --- | --- | --- | --- | --- | --- | --- | --- | --- | --- |
| ***How harmful do you think these are during pregnancy for your child’s brain and behavioral development (e.g., risk of ADHD, child's IQ, language skills, motor skills ...)?*** | | | | | | | | | | | | |
| Antidepressants (e.g., Zoloft, Fluoxetin, Cipralex, Seroxat) | **□** | **□** | **□** | **□** | **□** | **□** | **□** | **□** | **□** | **□** | **□** | **□** |
| Cranberry | **□** | **□** | **□** | **□** | **□** | **□** | **□** | **□** | **□** | **□** | **□** | **□** |
| Maternal psychiatric problem/disorder in itself | **□** | **□** | **□** | **□** | **□** | **□** | **□** | **□** | **□** | **□** | **□** | **□** |
| Alcohol (*e.g., wine, beer, spirits*) | **□** | **□** | **□** | **□** | **□** | **□** | **□** | **□** | **□** | **□** | **□** | **□** |
| ***How dangerous do you think these are during pregnancy in relation to the risk of miscarriage?*** | | | | | | | | | | | | |
| Antidepressants (e.g., Zoloft, Fluoxetin, Cipralex, Seroxat) | **□** | **□** | **□** | **□** | **□** | **□** | **□** | **□** | **□** | **□** | **□** | **□** |
| Cranberry | **□** | **□** | **□** | **□** | **□** | **□** | **□** | **□** | **□** | **□** | **□** | **□** |
| Maternal psychiatric problem/disorder in itself | **□** | **□** | **□** | **□** | **□** | **□** | **□** | **□** | **□** | **□** | **□** | **□** |
| Alcohol (*e.g., wine, beer, spirits*) | **□** | **□** | **□** | **□** | **□** | **□** | **□** | **□** | **□** | **□** | **□** | **□** |
| ***How dangerous do you think these are during pregnancy related to the child's risk of birth defects?*** | | | | | | | | | | | | |
| Antidepressants (e.g., Zoloft, Fluoxetin, Cipralex, Seroxat) | **□** | **□** | **□** | **□** | **□** | **□** | **□** | **□** | **□** | **□** | **□** | **□** |
| Cranberry | **□** | **□** | **□** | **□** | **□** | **□** | **□** | **□** | **□** | **□** | **□** | **□** |
| Maternal psychiatric problem/disorder in itself | **□** | **□** | **□** | **□** | **□** | **□** | **□** | **□** | **□** | **□** | **□** | **□** |
| Alcohol (*e.g., wine, beer, spirits*) | **□** | **□** | **□** | **□** | **□** | **□** | **□** | **□** | **□** | **□** | **□** | **□** |

***YOUR VIEWS ABOUT MEDICATIONS***

**We would like to ask you about your personal views about medicines. There are no right or wrong answers. We are interested in your personal thoughts.**

| 1. **Below is a list of some common views and assertions. Please specify how much you agree or disagree with them by ticking where appropriate. (You may only tick once per line)** | | | | | |
| --- | --- | --- | --- | --- | --- |
|  | Strongly agree | Agree | Uncertain | Disagree | Strongly disagree |
| 1. **All medicines can be harmful to the fetus.** | □ | □ | □ | □ | □ |
| 1. **Even if I’m ill, I believe it’s better for the fetus that I refrain from using medicines during pregnancy.** | □ | □ | □ | □ | □ |
| 1. **I have a higher threshold for using medicines when pregnant than when I’m not pregnant.** | □ | □ | □ | □ | □ |
| 1. **Thanks to treatment with medicines during pregnancy, the lives of many unborn children are saved each year.** | □ | □ | □ | □ | □ |
| 1. **It is better for the fetus that I use medicines and get well than to have an untreated illness during pregnancy.** | □ | □ | □ | □ | □ |
| 1. **Doctors prescribe too many medicines to pregnant women.** | □ | □ | □ | □ | □ |

***YOUR NEEDS FOR INFORMATION***

**Finally, there are some questions about your information needs.**

| 1. **From what sources did you get information about antidepressants during your pregnancy? (*You may tick more than one answer*)**   □ Family/friends  □ Physician  □ Midwife/Nurse  □ Pharmacy personnel  □ Internet (e.g., Google, pregnancy forums)  □ Media, including social media (e.g., Facebook)  □ Medically related websites (e.g., trygmammamedisin.no, nhi.no)  □ Staff at the health food store  □ Felleskatalog/package leaflet  □ Other (please specify: _______) |
| --- |
| 1. **If there were discrepancies among the sources, what did it mean to you? (*You may tick more than one answer*)**   □ There were no discrepancies among the sources  □ Nothing  □ I became anxious  □ I decided not to use the medication  □ I sought a new information source  □ I chose to rely on one source and ignore the conflicting one  □ I got information about antidepressants only from one source  □ Other |
| 1. **How often do you have difficulty understanding your health/medical condition or your medications due to difficulty understanding written information?**  - Very often - Often - Sometimes - Occasionally - Never - I don’t know  1. **How did you hear about this study?**  - Helseoversikt - Facebook - Tryggmammamedisin.no - Ammehjelp.no - Landsforening 1001 dager - Other, specify: ________ |

1. **Would you like to share any other comment/experience**?

________________________________________________________________________________________________________________________________________________________________________________________________________________________________________________________________________________________________________________________________________

##

## Appendix 2: STROBE Statement—checklist of items that should be included in reports of observational studies

|  | **Item No.** | **Recommendation** | **Page  No.** | **Relevant text from manuscript** |
| --- | --- | --- | --- | --- |
| **Title and abstract** | 1 | (*a*) Indicate the study’s design with a commonly used term in the title or the abstract | P 1 | “cross-sectional study” |
|  |  | (*b*) Provide in the abstract an informative and balanced summary of what was done and what was found | P 1 |  |
| **Introduction** | | | |  |
| Background/rationale | 2 | Explain the scientific background and rationale for the investigation being reported | P 1 |  |
| Objectives | 3 | State specific objectives, including any prespecified hypotheses | P 2, 3 | “This study aimed to…”  “We hypothesized that …” |
| **Methods** | | | |  |
| Study design | 4 | Present key elements of study design early in the paper | P 2 |  |
| Setting | 5 | Describe the setting, locations, and relevant dates, including periods of recruitment, exposure, follow-up, and data collection | P 2 |  |
| Participants | 6 | (*a*) *Cohort study*—Give the eligibility criteria, and the sources and methods of selection of participants. Describe methods of follow-up  *Case-control study*—Give the eligibility criteria, and the sources and methods of case ascertainment and control selection. Give the rationale for the choice of cases and controls  *Cross-sectional study*—Give the eligibility criteria, and the sources and methods of selection of participants | P 2, 4 | “Study Design and Data Collection”  “Figure 1” |
|  |  | (*b*) *Cohort study*—For matched studies, give matching criteria and number of exposed and unexposed  *Case-control study*—For matched studies, give matching criteria and the number of controls per case |  |  |
| Variables | 7 | Clearly define all outcomes, exposures, predictors, potential confounders, and effect modifiers. Give diagnostic criteria, if applicable | P 2, 3 |  |
| Data sources/ measurement | 8* | For each variable of interest, give sources of data and details of methods of assessment (measurement). Describe comparability of assessment methods if there is more than one group | P 2, 3 |  |
| Bias | 9 | Describe any efforts to address potential sources of bias | / |  |
| Study size | 10 | Explain how the study size was arrived at | P 2, 4 | “The target sample size…”  “Figure 1” |

| Quantitative variables | 11 | Explain how quantitative variables were handled in the analyses. If applicable, describe which groupings were chosen and why | P 3 | “To examine our first hypothesis” |
| --- | --- | --- | --- | --- |
| Statistical methods | 12 | (*a*) Describe all statistical methods, including those used to control for confounding | P 2, 3 |  |
|  |  | (*b*) Describe any methods used to examine subgroups and interactions | / |  |
|  |  | (*c*) Explain how missing data were addressed | P 4 | “Figure 1” |
|  |  | (*d*) *Cohort study*—If applicable, explain how loss to follow-up was addressed  *Case-control study*—If applicable, explain how matching of cases and controls was addressed  *Cross-sectional study*—If applicable, describe analytical methods taking account of sampling strategy | / |  |
|  |  | (*e*) Describe any sensitivity analyses | / |  |
| **Results** | | | | |
| Participants | 13* | (a) Report numbers of individuals at each stage of study—eg numbers potentially eligible, examined for eligibility, confirmed eligible, included in the study, completing follow-up, and analysed | P 4, 5, 6, s28 | “Table 4”  “Table 5”  “Figure 1”  “Figure S1” |
|  |  | (b) Give reasons for non-participation at each stage | P 4, 5, 6, s28 | “Abbreviations and notations” |
|  |  | (c) Consider use of a flow diagram | P 4 | “Figure 1” |
| Descriptive data | 14* | (a) Give characteristics of study participants (eg demographic, clinical, social) and information on exposures and potential confounders | P 3, 5 | “Study population characteristics”  “Table 1” |
|  |  | (b) Indicate number of participants with missing data for each variable of interest | P 4, 6, s28 | “Table 5”  “Figure 1”  “Figure S1” |
|  |  | (c) *Cohort study*—Summarise follow-up time (eg, average and total amount) |  |  |
| Outcome data | 15* | *Cohort study*—Report numbers of outcome events or summary measures over time |  |  |
|  |  | *Case-control study—*Report numbers in each exposure category, or summary measures of exposure |  |  |
|  |  | *Cross-sectional study—*Report numbers of outcome events or summary measures | P 4, 5, s25 | “Table 4”  “Table 5”  “Table S4: Distribution of EPDS, GAD-7 and MAMP-AD scores during pregnancy by trimester and postpartum” |
| Main results | 16 | (*a*) Give unadjusted estimates and, if applicable, confounder-adjusted estimates and their precision (eg, 95% confidence interval). Make clear which confounders were adjusted for and why they were included | P 4, 5, 6, s28 | “Table 4”  “Table 5”  “Figure S1” |
|  |  | (*b*) Report category boundaries when continuous variables were categorized | P 3, 4 | “To examine our first hypothesis…”  “EPDS score ≥13” “at least six of the past seven days” “score >7.5 out of 10” |
|  |  | (*c*) If relevant, consider translating estimates of relative risk into absolute risk for a meaningful time period | / |  |

| Other analyses | 17 | Report other analyses done—eg analyses of subgroups and interactions, and sensitivity analyses | / |  |
| --- | --- | --- | --- | --- |
| **Discussion** | | | | |
| Key results | 18 | Summarise key results with reference to study objectives | P 4 |  |
| Limitations | 19 | Discuss limitations of the study, taking into account sources of potential bias or imprecision. Discuss both direction and magnitude of any potential bias | P 6 |  |
| Interpretation | 20 | Give a cautious overall interpretation of results considering objectives, limitations, multiplicity of analyses, results from similar studies, and other relevant evidence | P 4, 5, 6 |  |
| Generalisability | 21 | Discuss the generalisability (external validity) of the study results | P 6 | “This could limit the generalizability of findings…” |
| **Other information** | |  | | |
| Funding | 22 | Give the source of funding and the role of the funders for the present study and, if applicable, for the original study on which the present article is based | / |  |

*Give information separately for cases and controls in case-control studies and, if applicable, for exposed and unexposed groups in cohort and cross-sectional studies.

**Note:** An Explanation and Elaboration article discusses each checklist item and gives methodological background and published examples of transparent reporting. The STROBE checklist is best used in conjunction with this article (freely available on the Websites of PLoS Medicine at http://www.plosmedicine.org/, Annals of Internal Medicine at http://www.annals.org/, and Epidemiology at http://www.epidem.com/). Information on the STROBE Initiative is available at www.strobe-statement.org.

## Table S1: Overview of studies on adherence to antidepressants in pregnancy

| **Author and year** | **Details on the study design and population** | **How adherence was measured** | **Rate of low/high adherence** | **Comment** |
| --- | --- | --- | --- | --- |
| [Lupattelli A. et al., 2015](https://doi.org/10.1002/da.22352) ^1^ | Multinational, cross-sectional, web-based study; Data were collected between October 1, 2011, and February 29, 2012; Subjects: pregnant women who reported use of psychotropic medications (n=160) | Validated self-report medication adherence measure (**MMAS-8**) | Low adherence:  48.8%, (n=78)  95% CI: 41.1%–56.4%  medication for anxiety: 51.3%  medication for depression: 47.2% medication for other psychiatric disorders: 42.9% | Almost 50% of women demonstrated low adherence to psychotropic medication during pregnancy. |
| [Bosman J et al., 2014](https://doi.org/10.1177/2045125313511486) ^2^ | An observational study, performed in an outpatient population from January 2010 until January 2012, 41 women were included during the first trimester of pregnancy; data could be evaluated in 29 women. Pill count, BMQ, and TDM were evaluated against the standard, MEMS | % **Pill count:** number of prescribed pills − number of pills left in the bottle)/(number of days between dispensing date and return date of a pill bottle) × 100.  The cut-off point for good adherence was ≥90%. | The median percentage of pills taken was 99%, with a range of 72.2%–103.9%  Adherent:   93% of women | Compared with MEMS, only the pill count had a good agreement for adherence.    TDM and BMQ were not associated with MEMS and were not appropriate methods to measure this pregnant population's adherence.  It seems that pill counts can be used instead of MEMS in daily practice.  Adherence to ADs during pregnancy was relatively high, compared with data from nonpregnant women with chronic medication use or the general population with antidepressants.^3-5^ |
|  |  | Self-reported questionnaire **BMQ** was evaluated at the end of the study period and was used to measure the perception of the use of antidepressants. | **BMQ:**  Adherent:  65% of pregnant women  Adherent groups: Acceptors: 48.3%, Ambivalent:17.2%  Poor adherence: Indifferent: 31.0% Skeptical: 3.4% |  |
|  |  | **TDM**  Every trimester and 2–3 months post-partum, the blood concentration of the antidepressant was measured for possible relationships with adherence. | **TDM:**  75% were adherent (samples were within the therapeutic range) |  |
|  |  | **MEMS** percentage of doses taken on schedule within 25% of the expected time interval. The cut-off point for good adherence was ≥80% | **MEMS:**  86% of women were adherent (took more than 80% of all doses on schedule) |  |
| [Adhikari K. et al., 2018](https://doi-org.ezproxy.uio.no/10.1177%2F0706743718802809) ^6^ | A retrospective cohort study, used population-based administrative data. The study population were women diagnosed with depression which gave birth between 2012 and 2015 and were adherent (MPR≥80%) to ≥2 consecutive antidepressant prescriptions during the preconception year (n = 1865) | **Adherence** is measured with **MPR,** which assessed the proportion of a given period where medication supply was available. Women were classified as adherent if the MPR was ≥80%.  **Persistence** (continuity of prescribed treatment over several prescriptions) was calculated at each month of pregnancy; Women were classified as persistent if the difference was ≤30 days when subtracting the duration of pregnancy period from the sum of the total supply of medication dispensed between the first refill date to the second last refill date. | **Discontinuation in pregnancy**  44.7% (95% CI, 42.4% to 47.0%)  **SNRIs or SSRIs:** 45.0%  **NSMRIs:** 100.0%  **Atypical antidepressants:** 73.8%  **Adherence during pregnancy**  62.6% (95% CI, 59.4% to 65.7%)  **SNRIs**  75.1% (95% CI, 68.3% to 80.9%)  **SSRIs**  60.9% (95% CI, 57.2% to 64.5%)  **NSMRIs**  42.9% (95% CI, 19.9% to 69.3%)  **Atypical antidepressants:**  37.5% (95% CI, 22.5% to 55.4%) | Persistence to ADs substantially decreased as the duration of pregnancy progressed and was consistently higher for SNRIs in each pregnancy point compared with other antidepressant classes.  Only, 40.7% of women were persistent with antidepressants for the full pregnancy period |

**Abbreviations and notations:** **MMAS-8** – Eight-item Morisky Medication Adherence Scale; **BMQ** - Beliefs about Medicine Questionnaire; **MEMS** - Medication Event Monitoring System; **TDM** - Therapeutic Drug Monitoring; **MPR** - Medication Possession Ratio; **SSRIs** - Selective Serotonin Reuptake Inhibitors; **SNRIs -** Serotonin-Norepinephrine Reuptake Inhibitors; **NSMRIs** - Non-Selective Monoamine Reuptake Inhibitors; **AD** - antidepressants; **CI** - Confidence Interval.

##

## Table S2: The 16 MAMP-AD* scale items with their scientific rationale

| 1. **How often do you forget to take your antidepressant?** |
| --- |
| In the general population, in 30 percent of cases of not taking prescribed medication reason is forgetfulness.^7^ Most of the self-report adherence scales consider this fact and include one or more questions about forgetfulness.^8^ |
| 1. **Do you ever stop or skip a dose of your antidepressants when you feel better?** 2. **Do you ever reduce the dose of your antidepressant when you feel better? (For example, if you take one tablet instead of 2 tablets, or take ½ tablet instead of 1 tablet)** |
| The American Medical Association (AMA) names a lack of symptoms as one of the eight reasons patients don't take their medications.^9^ Once patients feel better, they may think the problem has resolved and may discontinue using the medicine, even if they need continuation of therapy. The belief that symptom severity does not justify medication is also one of the barriers to adherence in pregnancy.^10^  These two questions distinguish two types of adherence: skipping one or more doses and reducing the dose. After consulting associates in this project, we added examples of reducing the dose in brackets, making it more understandable for participants. |
| 1. **Do you ever use less antidepressants (than prescribed by the doctor) because you think it is safer for your unborn child?** |
| Women tend to overestimate the magnitude of drug’s teratogenic risks^11^ and consider AD to be harmful in pregnancy almost as much as alcohol or smoking.^12^ Focus groups research from the “HEALTHx2” project^13^ finds that pregnant women often take less medication than a doctor has prescribed, hoping that a lower quantity of the medication will enter the fetus’s bloodstream. The lack of knowledge (uncertainty) around prescribing antidepressants, difficulty prioritizing women/children, guilt because women need to take antidepressants, and fear of adverse effects make women think that lower doses produce less adverse effects. Skipping, stopping, and reducing the dose are united in “using less antidepressants” in the final version of the scale. |
| 1. **Do you ever avoid taking antidepressants (as prescribed by the doctor) because they do not improve your symptoms sufficiently?** |
| Some patients do not take their medicines because they believe that they are not working.^14^ In some cases, non-adherence can happen when a patient does not understand the time it takes to see results.^9^ |
| 1. **Do you ever avoid taking antidepressants (as prescribed by the doctor) because they make you feel emotionally blunt and less like yourself?** |
| One of the barriers to antidepressant treatment discovered in the focus group interviews in the “HEALTHx2” project^13^ is that women often experienced flattening their emotions following antidepressant treatment. That made them fear that they would not bond with the child or be good enough mothers. «Emotional bluntness» is one of the side effects of antidepressants that is especially challenging in pregnancy,^13^ where the mother-child bond is established. |
| 1. **Do you sometimes not take your antidepressants (as prescribed by the doctor) because financial or practical reasons limit your access to them?** |
| The cost of the medicine prescribed could be a significant barrier to adherence.^9, 15^ The high price may lead to patients not filling their medications or rationing what they do fill in order to extend their supply. Health care in Norway is such that the price of medication is usually not a problem for the patient. However, other practical issues can lead to non-adherence, such as long waitlists for the doctor or distance from health services. This question was included so that the scale could be used internationally. |
| 1. **Do you ever avoid taking your antidepressants (as prescribed by the doctor) because you prioritize taking other medications?** |
| Taking too many medications could be a barrier to adherence.^9^ Having several different medicines prescribed with higher dosing frequency increases the chances of being non-adherent. Another aspect of polypharmacy in pregnancy is the fear of the adverse effects of different medications on the fetus. A woman could exclude one medicine from the therapy because she considers the other drug more essential and doesn’t want her child to be affected by both. |
| 1. **Do you ever avoid taking your antidepressants (as prescribed by the doctor) because they give you too many side effects?** |
| Fear of experiencing previous side effects with the same or similar medicine is a significant barrier to non-adherence.^9, 16^ |
| 1. **Do you take your antidepressants precisely as the doctor has prescribed?** |
| The 2003 report published by the WHO^4^ defines adherence as “the extent to which the patient follows medical instructions.” This question should capture the extent to which the patient considers that he is following the instructions given by the doctor. |
| 1. **Do you take your antidepressants only when you feel your symptoms are getting worse?**** |
| Once a patient’s condition is under control, they may think they can discontinue using the medication.^9^ However, sometimes the patient does not understand the need for prolonged medicine use, which often results in a relapse of illness.^17^ |
| 1. **Are the benefits of taking antidepressants greater than the disadvantages for you?** |
| This question should assess women’s perception of the risk-benefit ratio connected to antidepressant use. Greater symptom severity, positive attitude toward antidepressants, and acceptance and understanding of mental illness are factors that promote adherence in both general and perinatal populations.^10^ |
| 1. **Do you believe that taking antidepressants will enable you to take better care of your child once it is born?** |
| Focus group interviews in the HEALTHx2^13^ study have found that being functional and taking care of the child and family are important motivating factors for taking antidepressants during pregnancy. |
| 1. **Do you believe that taking antidepressants can prevent disease relapse during pregnancy and/or after birth?** |
| HEALTHx2 focus group interviews^13^ detected that fear of relapse, especially after birth, is the key to high antidepressant adherence. Pregnant women might fear getting sick again when their child needs them the most. For women with a history of severe or recurrent depression, this fear is well-founded since a meta-analysis finds that women with such morbidities have more than doubled the risk of relapse after discontinuation of antidepressants (RR=2.30).^18^ |
| 1. **Do you believe that not treating your disorder with antidepressants during pregnancy may be harmful to your unborn child?** |
| As detected in the HEALTHx2 focus group interviews, having a good quality of life, being functional, and taking care of the child/family are motivating factors for taking antidepressants. Not taking them could endanger the mother’s euthymia and lead to risky behavior in pregnancy, such as smoking or drinking alcohol, which further threatens the unborn child’s development. |
| 1. **When it comes to your treatment with antidepressants during pregnancy, do you trust the doctor's judgment?** |
| One of the barriers to medication adherence during pregnancy is the lack of an optimal patient-physician relationship.^15^ Mistrust can make the patient suspicious of the doctor’s motives for prescribing certain medications, particularly in light of marketing efforts by pharmaceutical companies influencing physician prescribing patterns.^9^ |

**Notation:** **(*)** The first nine MAMP-AD items used a five-point Likert scale to assess the frequency of behaviors, with response options ranging from "very often" to "never" and scores from 0 to 4. The last seven items had response options of "no," "uncertain," and "yes," scored from 0 to 2. **(**)** The scoring for *Item 11* is reversed.

## Table S3: Antidepressants used in the study population (n=90)

| Antidepressant | n (%) |
| --- | --- |
| Escitalopram | 41 (45.6) |
| Sertraline | 24 (26.7) |
| Venlafaxine | 6 (6.7) |
| Fluoxetine | 5 (5.6) |
| Vortioxetine | 5 (5.6) |
| Citalopram | <5 |
| Paroxetine | <5 |
| Mirtazapine | <5 |
| Amitriptyline | <5 |
| Bupropion | <5 |

## Table S4: Distribution of EPDS, GAD-7 and MAMP-AD scores during pregnancy by trimester and postpartum

|  |  | EPDS | | | GAD-7 | | | MAMP-AD | |
| --- | --- | --- | --- | --- | --- | --- | --- | --- | --- |
|  | **N** | **Mean**  **(sd)** | **Median**  **(IQR)** | **≥13 points cutoff**  **n (%)** | **Mean**  **(sd)** | **Median**  **(IQR)** | **≥10 points cutoff**  **n (%)** | **Mean**  **(sd)** | **Median**  **(IQR)** |
| Pregnancy status | | | | | | | | | |
| Pregnant* | 49 | 9.7 (4.9) | 9.0 (5-14) | 15 (30.6) | 7.2 (4.3) | 7.0 (4-10) | 14 (28.6) | 44.6 (4.8) | 46.0 (44.8-47) |
| 1^st^ trimester | 13 | 9.2 (5.0) | 9.0 (5-14) | 4 (30.8) | 7.9 (4.9) | 7.0 (4-11) | 5 (38.5) | 44.8 (4.7) | 46.0 (44.8-48) |
| 2^nd^ trimester** | 17 | 11.0 (4.9) | 11.0 (7-14) | 6 (35.3) | 7.9 (3.9) | 7.0 (6-9) | 4 (23.5) | 44.9 (4.8) | 46.0 (45-47) |
| 3^rd^ trimester | 18 | 8.6 (4.8) | 8.0 (4-12) | 4 (22.2) | 6.1 (4.3) | 5.5 (2-10) | 5 (27.8) | 44.4 (5.0) | 46.5 (43-47) |
| Postpartum | 41 | 7.6 (4.8) | 6.0 (5-12) | 10 (24.4) | 6.9 (4.6) | 6.0 (4-8) | 6 (14.6) | 44.6 (4.8) | 47.0 (44-48) |

**Abbreviations and notations:** **EPDS** - Edinburgh Perinatal Depression Scale (score range 0-30); **GAD-7** - Generalized Anxiety Disorder Scale (score range 0-21); **MAMP-AD** - Medication Adherence Measurement in Pregnancy for Antidepressants (score range 0-48); **sd** - standard deviation; **IQR** - Interquartile Range, presented as the range from the 25th percentile to the 75th percentile; (*****) One pregnant participant did not indicate trimester; (******)Two pregnant paricipants in the second trimester did not answer all the questions from the EPDS.

## Table S5: Exploratory factor analysis - Rotated factor loading matrix* and unique variances** for all 16 MAMP-AD scale items, providing the rational for excluding item 15 (low factor loading, high uniqueness)

| Variable | Factor 1 | Factor 2 | Factor 3 | Factor 4 | Uniqueness |
| --- | --- | --- | --- | --- | --- |
| ITEM 1 |  |  |  | 0.64 | 0.52 |
| ITEM 2 | 0.84 |  |  |  | 0.24 |
| ITEM 3 | 0.83 |  |  |  | 0.22 |
| ITEM 4 | 0.58 |  |  |  | 0.43 |
| ITEM 5 |  |  | 0.65 |  | 0.56 |
| ITEM 6 |  |  | 0.70 |  | 0.50 |
| ITEM 7 |  |  |  | 0.71 | 0.42 |
| ITEM 8 |  |  |  | 0.43 | 0.56 |
| ITEM 9 |  |  | 0.62 |  | 0.52 |
| ITEM 10 | 0.52 | 0.67 |  |  | 0.25 |
| ITEM 11 | 0.81 |  |  |  | 0.21 |
| ITEM 12 |  | 0.65 |  |  | 0.54 |
| ITEM 13 |  | 0.48 |  |  | 0.72 |
| ITEM 14 |  | 0.52 |  |  | 0.60 |
| ITEM 15 |  |  |  |  | 0.84 |
| ITEM 16 | 0.45 |  |  |  | 0.67 |

**Abbreviations and notations:** **MAMP-AD** - Medication Adherence Measurement in Pregnancy for Antidepressants; **(*)** Loadings lower than 0.4 are blanked. **(**)** Item uniqueness represents each item's specific and error variance, calculated as 1 minus the total variance explained by all factors.

## Table S6: Summary statistics: The distributional properties of individual items in the MAMP-AD scale. Data from the exploratory factor analysis (EFA).

|  | MAMP-AD items | | | | | | | | | | | | | | | | |
| --- | --- | --- | --- | --- | --- | --- | --- | --- | --- | --- | --- | --- | --- | --- | --- | --- | --- |
| Scale item | **1** | **2** | **3** | **4** | **5** | **6** | **7** | **8** | **9** | **10** | **11R*** | **12** | **13** | **14** | **15** | **16** |  |
| Mean | 3.39 | 3.70 | 3.66 | 3.53 | 3.97 | 3.92 | 3.90 | 3.92 | 3.84 | 1.84 | 1.98 | 1.81 | 1.63 | 1.69 | 1.29 | 1.80 |  |
| Median | 4 | 4 | 4 | 4 | 4 | 4 | 4 | 4 | 4 | 2 | 2 | 2 | 2 | 2 | 1.5 | 2 |  |
| Sd | 0.77 | 0.88 | 0.95 | 1.21 | 0.18 | 0.34 | 0.43 | 0.37 | 0.47 | 0.52 | 0.15 | 0.45 | 0.64 | 0.61 | 0.80 | 0.45 |  |
| Minimum | 0 | 0 | 0 | 0 | 3 | 2 | 1 | 2 | 2 | 0 | 1 | 0 | 0 | 0 | 0 | 0 |  |
| Maximum | 4 | 4 | 4 | 4 | 4 | 4 | 4 | 4 | 4 | 2 | 2 | 2 | 2 | 2 | 2 | 2 |  |
| Skewness | -1.67 | -3.16 | -2.82 | -2.33 | -5.20 | -4.64 | -5.00 | -4.76 | -3.05 | -3.14 | -6.48 | -2.32 | -1.52 | -1.80 | -0.56 | -2.19 |  |
| Kurtosis | 6.92 | 12.33 | 9.89 | 6.67 | 28.03 | 24.27 | 30.00 | 24.18 | 11.31 | 11.18 | 43.02 | 7.75 | 4.02 | 4.93 | 1.81 | 7.12 |  |

**Abbreviations and notations:** **MAMP-AD** - Medication Adherence Measurement in Pregnancy for Antidepressants; **sd** – standard deviation; (*) The scoring for *Item 11* is reversed

##

## Table S7: Exploratory factor analysis based on Spearman correlations - rotated factor loading matrix* and unique variances** for 14 MAMP‑AD items***

| Variable | Factor 1 | Factor 2 | Factor 3 | Uniqueness |
| --- | --- | --- | --- | --- |
| ITEM 2 | 0.71 |  |  | 0.43 |
| ITEM 3 | 0.79 |  |  | 0.34 |
| ITEM 4 | 0.60 | 0.48 |  | 0.37 |
| ITEM 5 |  |  | 0.64 | 0.57 |
| ITEM 6 |  |  | 0.72 | 0.49 |
| ITEM 7 | 0.41 |  | 0.44 | 0.63 |
| ITEM 8 | 0.48 |  |  | 0.55 |
| ITEM 9 |  |  | 0.65 | 0.48 |
| ITEM 10 |  | 0.76 |  | 0.31 |
| ITEM 11 | 0.44 | 0.48 |  | 0.56 |
| ITEM 12 |  | 0.45 |  | 0.72 |
| ITEM 13 |  | 0.53 |  | 0.70 |
| ITEM 14 |  | 0.53 |  | 0.62 |
| ITEM 16 |  | 0.48 |  | 0.69 |

**Abbreviations and notations:** **MAMP-AD** - Medication Adherence Measurement in Pregnancy for Antidepressants; **(*)** Loadings lower than 0.4 are blanked. **(**)** Item uniqueness represents each item's specific and error variance, calculated as 1 minus the total variance explained by all factors.(*******)Third EFA after excluding item 15 (round 1) and item 1 (round 2) for low loadings/high uniqueness

## Figure S1: Construct validity: association between antidepressant adherence (MAMP-AD score*) and depressive symptoms (EDPS) (A), number of days in a week antidepressants were taken exactly as prescribed (B) and perceived antidepressant benefit (C)

| **(A)**  Each unit increase in the MAMP-AD score was associated with 26% lower odds of having clinically relevant depressive symptoms (≥13 EPDS score).   - OR (95%CI) = 0.74 (0.56, 0.99) - AUC = 0.73 - n = 36** | 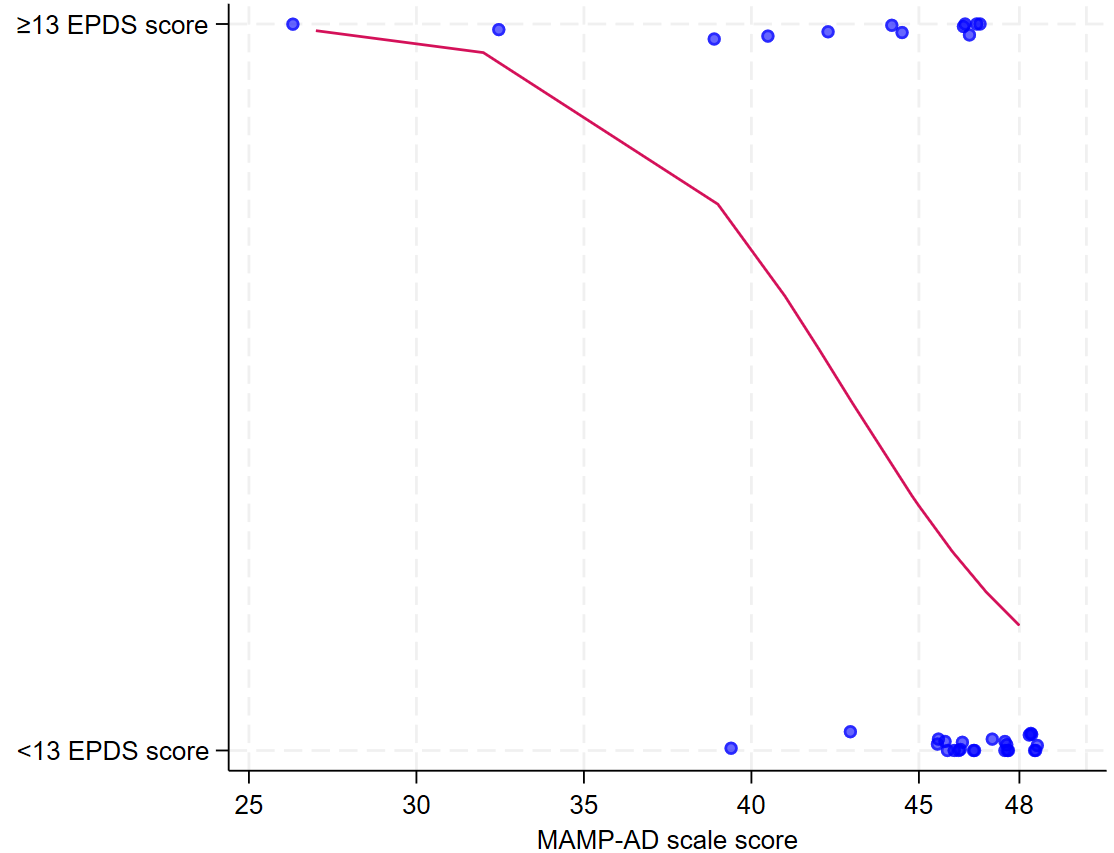 |
| --- | --- |
| **(B)**  Each unit increase in the MAMP-AD score was associated with 32% higher odds of being adherent to the treatment at least 80% of the time (≥ 6 of 7 weekdays).   - OR (95% CI) = 1.32 (1.13, 1.54) - AUC = 0.93 - n = 82****** | 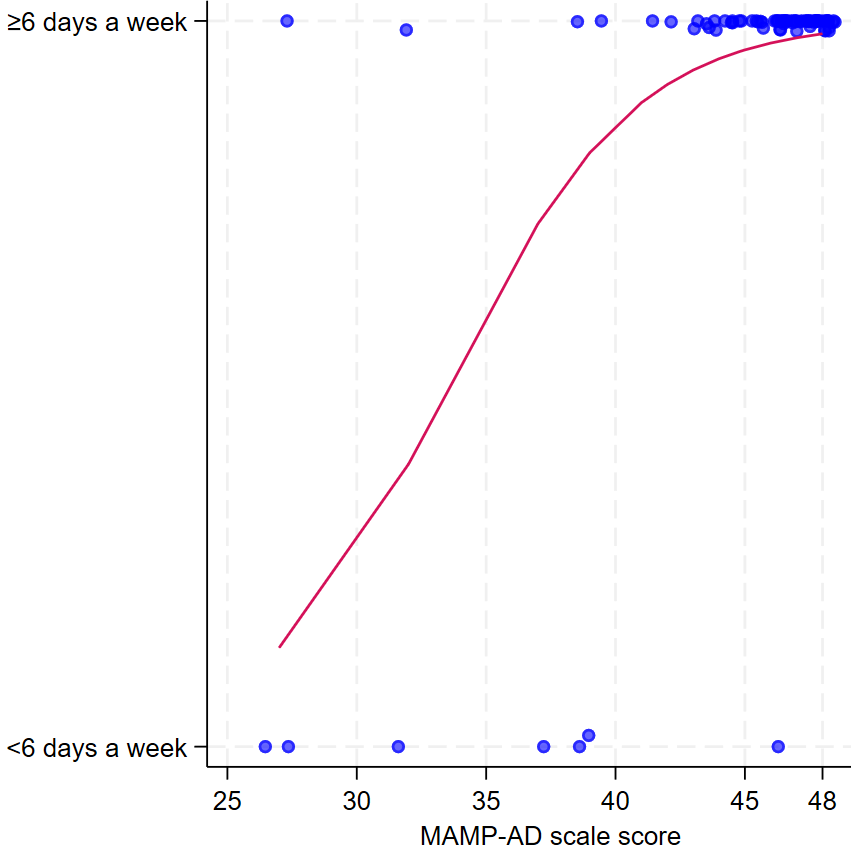 |
| **(C)**  Each unit increase in the MAMP-AD score was associated with a 26% higher odds of perceiving antidepressants as beneficial (≥ 7.5 on a 0–10 scale).   - OR (95%CI) = 1.26 (1.10, 1.43) - AUC = 0.83 - n = 88****** | 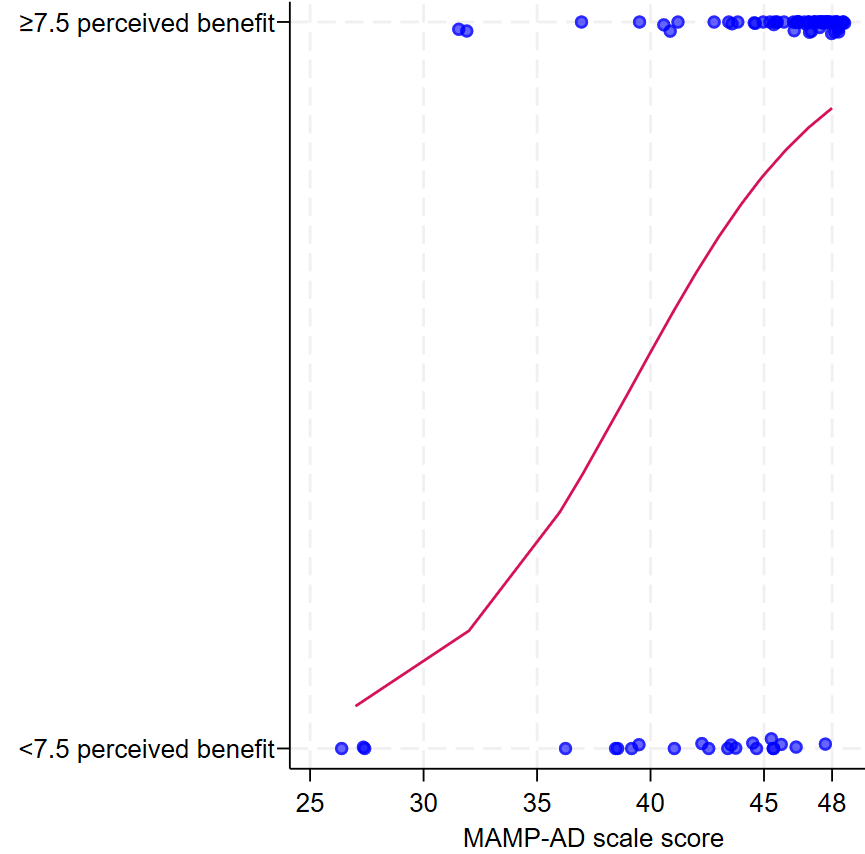 |

**Abbreviations and notations:** **MAMP-AD** - Medication Adherence Measurement in Pregnancy for Antidepressants; **EPDS** - Edinburgh Perinatal Depression Scale (score 0-30); **OR** – Odds ratio; **CI** - Confidence interval; **AUC** - Area under the curve; **(*)** The final MAMP-AD scale consisted of 15 items, scoring from 0 to 48. **(**)** Out of 38 pregnant participants using antidepressants at the time of questionnaire completion, 36 responded to all questions on the EPDS. Of the total 90 participants, 82 answered the question regarding the number of days they took antidepressants exactly as prescribed in the past week, while 88 responded to the question about the perceived benefits of their antidepressant treatment

## References

1. Lupattelli A, Spigset O, Björnsdóttir I, Hämeen-Anttila K, Mårdby A-C, Panchaud A, et al. Patterns and factors associated with low adherence to psychotropic medications during pregnancy - a cross-sectional, multinational web-based study. Depress Anxiety. 2015;32(6):426-36. <https://doi.org/10.1002/da.22352>

2. Bosman J, ter Horst PGJ, Smit JP, Dijkstra JR, Beekhuis HR, Slingersland RJ, Hospes W. Adherence of antidepressants during pregnancy: MEMS compared with three other methods. Ther Adv Psychopharmacol. 2014;4(2):61-9. <https://doi.org/10.1177/2045125313511486>

3. Sawicki E, Stewart K, Wong S, Leung L, Paul E, George J. Medication use for chronic health conditions by pregnant women attending an Australian maternity hospital. Aust N Z J Obstet Gynaecol. 2011;51(4):333-8. <https://doi.org/10.1111/j.1479-828X.2011.01312.x>

4. World Health Organization. Adherence to long-term therapies: evidence for action. Geneva: World Health Organization; 2003. <https://iris.who.int/handle/10665/42682>

5. Muzina DJ, Malone DA, Bhandari I, Lulic R, Baudisch R, Keene M. Rate of non-adherence prior to upward dose titration in previously stable antidepressant users. J Affect Disord. 2010;130(1):46-52. <https://doi.org/10.1016/j.jad.2010.09.018>

6. Adhikari K, Patten SB, Lee S, Metcalfe A. Adherence to and Persistence with Antidepressant Medication during Pregnancy: Does It Differ by the Class of Antidepressant Medication Prescribed? Can J Psychiatry. 2019;64(3):199-208. <https://doi.org/10.1177/0706743718802809>

7. Osterberg L, Blaschke T. Drug therapy - Adherence to medication. N Engl J Med. 2005;353(5):487-97. <https://doi.org/10.1056/NEJMra050100>

8. Nguyen T-M-U, Caze AL, Cottrell N. What are validated self-report adherence scales really measuring?: a systematic review. Br J Clin Pharmacol. 2014;77(3):427-45. <https://doi.org/https://doi.org/10.1111/bcp.12194>

9. AMA_Physician_Communications_team. 8 reasons patients don't take their medications [updated Feb 22, 2023 Available from: <https://www.ama-assn.org/delivering-care/physician-patient-relationship/8-reasons-patients-dont-take-their-medications>.

10. Misri S, Eng AB, Abizadeh J, Blackwell E, Spidel A, Oberlander TF. Factors impacting decisions to decline or adhere to antidepressant medication in perinatal women with mood and anxiety disorders. Depress Anxiety. 2013;30(11):1129-36. <https://doi.org/10.1002/da.22137>

11. Mulder B, Bijlsma MJ, Schuiling-Veninga CC, Morssink LP, van Puijenbroek E, Aarnoudse JG, et al. Risks versus benefits of medication use during pregnancy: What do women perceive? Patient Prefer Adherence. 2018;12:1-8. <https://doi.org/10.2147/PPA.S146091>

12. Petersen I, McCrea RL, Lupattelli A, Nordeng H. Women's perception of risks of adverse fetal pregnancy outcomes: a large-scale multinational survey. BMJ Open. 2015;5(6):e007390-e. <https://doi.org/10.1136/bmjopen-2014-007390>

13. Tauqeer F, Moen A, Myhr K, Wilson CA, Lupattelli A. Assessing decisional conflict and challenges in decision-making among perinatal women using or considering using antidepressants during pregnancy—a mixed-methods study. Arch Womens Ment Health. 2023;26(5):669-83. <https://doi.org/10.1007/s00737-023-01341-0>

14. Lin EHB, Korff MV, Katon W, Bush T, Simon GE, Walker E, Robinson P. The Role of the Primary Care Physician in Patients' Adherence to Antidepressant Therapy. Med Care. 1995;33(1):67-74. <https://doi.org/10.1097/00005650-199501000-00006>

15. Oladejo M, Bewley S. ADHERENCE IN PREGNANCY: A SYSTEMATIC REVIEW OF THE LITERATURE. Fet Matern Med Rev. 2012;23(3-4):201-29. <https://doi.org/10.1017/S0965539512000113>

16. Hung C-I, Wang S-J, Liu C-Y, Hsu S-C, Yang C-H. Comorbidities and factors related to discontinuation of pharmacotherapy among outpatients with major depressive disorder. Compr Psychiatry. 2011;52(4):370-7. <https://doi.org/10.1016/j.comppsych.2010.08.005>

17. Cohen LS, Altshuler LL, Harlow BL, Nonacs R, Newport DJ, Viguera AC, et al. Relapse of Major Depression During Pregnancy in Women Who Maintain or Discontinue Antidepressant Treatment. JAMA. 2006;295(5):499-507. <https://doi.org/10.1001/jama.295.5.499>

18. Bayrampour H, Kapoor A, Bunka M, Ryan D. The Risk of Relapse of Depression During Pregnancy After Discontinuation of Antidepressants: A Systematic Review and Meta-Analysis. J Clin Psychiatry. 2020;81(4). <https://doi.org/10.4088/JCP.19r13134>
